# Sequence elements within the PEXEL motif and its downstream region modulate PTEX‐dependent protein export in *Plasmodium falciparum*

**DOI:** 10.1111/tra.12922

**Published:** 2023-11-05

**Authors:** Mikha Gabriela, Claudia B. G. Barnes, Dickson Leong, Brad E. Sleebs, Molly Parkyn Schneider, Dene R. Littler, Brendan S. Crabb, Tania F. de Koning‐Ward, Paul R. Gilson

**Affiliations:** ^1^ Malaria Virulence and Drug Discovery Group Burnet Institute Melbourne Victoria Australia; ^2^ School of Medicine Deakin University Geelong Victoria Australia; ^3^ The Walter and Eliza Hall Institute of Medical Research Parkville Victoria Australia; ^4^ Department of Medical Biology The University of Melbourne Parkville Victoria Australia; ^5^ Infection and Immunity Program and Department of Biochemistry and Molecular Biology, Biomedicine Discovery Institute Monash University Clayton Victoria Australia; ^6^ Department of Microbiology and Immunology University of Melbourne Parkville Victoria Australia; ^7^ Department of Immunology Monash University Melbourne Victoria Australia; ^8^ Institute for Mental and Physical Health and Clinical Translation (IMPACT) Deakin University Geelong Victoria Australia

**Keywords:** chaperone, malaria, PEXEL, *Plasmodium falciparum*, protein export, PTEX, virulence

## Abstract

The parasite *Plasmodium falciparum* causes the most severe form of malaria and to invade and replicate in red blood cells (RBCs), it exports hundreds of proteins across the encasing parasitophorous vacuole membrane (PVM) into this host cell. The exported proteins help modify the RBC to support rapid parasite growth and avoidance of the human immune system. Most exported proteins possess a conserved *Plasmodium* export element (PEXEL) motif with the consensus RxLxE/D/Q amino acid sequence, which acts as a proteolytic cleavage recognition site within the parasite's endoplasmic reticulum (ER). Cleavage occurs after the P_1_ L residue and is thought to help release the protein from the ER so it can be putatively escorted by the HSP101 chaperone to the parasitophorous vacuole space surrounding the intraerythrocytic parasite. HSP101 and its cargo are then thought to assemble with the rest of a *Plasmodium* translocon for exported proteins (PTEX) complex, that then recognises the xE/D/Q capped N‐terminus of the exported protein and translocates it across the vacuole membrane into the RBC compartment. Here, we present evidence that supports a dual role for the PEXEL's conserved P_2_′ position E/Q/D residue, first, for plasmepsin V cleavage in the ER, and second, for efficient PTEX mediated export across the PVM into the RBC. We also present evidence that the downstream ‘spacer’ region separating the PEXEL motif from the folded functional region of the exported protein controls cargo interaction with PTEX as well. The spacer must be of a sufficient length and permissive amino acid composition to engage the HSP101 unfoldase component of PTEX to be efficiently translocated into the RBC compartment.

## Graphical abstract

In the malaria‐causing parasite *Plasmodium falciparum*, parasite proteins (left, red) are exported across an encasing parasitophorous vacuole membrane (PVM) (left, green) via the *Plasmodium* translocon for exported proteins (PTEX) whose core is comprised of HSP101, PTEX150 and EXP2 (right).

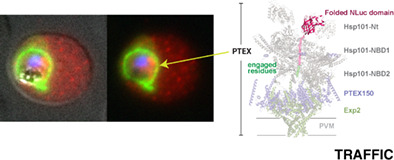

## 1 INTRODUCTION

The pathogenesis of malaria relies upon the ability of *Plasmodium* parasites, the causative agents of the disease, to propagate asexually within red blood cells (RBCs) while avoiding the immune mechanisms of their host. To accomplish this, the parasite exports hundreds of its proteins into its host cell to transform it into a hospitable niche and to avoid host immunity.^1^, ^2^, ^3^, ^4^, ^5^ The intraerythrocytic stage of the parasite resides within a membranous compartment called the parasitophorous vacuole (PV).^6^, ^7^ Consequently, parasite proteins destined for export must traverse two membranes, the parasite plasma membrane and the encasing parasitophorous vacuole membrane (PVM), to reach the host RBC. The *Plasmodium* translocon of exported proteins (PTEX) mediates the translocation of proteins across the PVM^8^ and is the only protein channel known to reside at the PVM. PTEX is comprised of three core components, HSP101, PTEX150 and EXP2, that are all essential for parasite survival, making it an attractive drug target candidate.^8^, ^9^, ^10^

Parasite proteins destined for export into the RBC begin their journey with entry into the endoplasmic reticulum (ER) via the Sec61 translocon, followed by cleavage of the protein within the ER by the aspartyl protease plasmepsin V (PMV).^11^, ^12^, ^13^ The cleavage step occurs within a pentameric amino acid motif near the N‐terminus of the protein destined for export, termed the *Plasmodium* export element (PEXEL).^1^, ^2^, ^14^ The resulting mature proteins then travel via the vesicular transport pathway to the parasite plasma membrane where they are secreted into the PV.^15^ Here, the exported proteins are unfolded and translocated across the PVM and into the host cell compartment by PTEX.^16^ It is thought the AAA+ ATPase HSP101 first engages and unfolds cargo proteins in an ATP‐dependent manner and threads them through a tetradecameric membrane‐spanning channel consisting of the scaffold protein PTEX150 and the pore‐forming protein EXP2.^17^, ^18^, ^19^ However, the information that is contained within proteins destined for export that allows HSP101 to specifically recognise these proteins prior to the unfolding step is still unknown.^20^, ^21^

Historically, the N‐terminal region of exported proteins (and the protein's transmembrane domain in some cases) has been found to be sufficient to mediate the trafficking of proteins into the RBC compartment.^22^, ^23^, ^24^, ^25^, ^26^ This N‐terminal region usually contains a recessed signal peptide to promote entry of the proteins into the parasite's ER^27^ or a hydrophobic stretch of amino acids that serves the same function.^28^ The discovery of the five‐amino acid PEXEL motif in the N‐terminal region of many exported proteins was the first export‐specific signal^1^, ^2^ that was proven to be predictive for exported proteins^29^ and allowed the identification of >450 putative exported proteins in *P. falciparum*.^4^ Interestingly, however, the presence of the PEXEL motif alone does not guarantee export, as its location within a protein's primary structure^30^ and the presence of ~12 amino acids downstream of the motif have also been shown to be required to achieve efficient export.^24^ Furthermore, PEXEL‐negative exported proteins (PNEPs) are also present in the *Plasmodium* exportome,^5^, ^31^, ^32^ suggesting that PEXEL is not strictly required for passage through PTEX.

The function of the PEXEL motif has been relatively well studied and its consensus sequence is RxLxE/D/Q where x can be almost any amino acid. The position P_3_ arginine (R) and P_1_ leucine (L) residues are the most conserved of the PEXEL motif and in *P. falciparum* have been shown to be necessary for efficient cleavage by PMV.^12^, ^13^, ^14^ Although most exported proteins utilise P_3_ R and P_1_ L residues some proteins utilise other amino acids with similar properties.^33^ It is assumed therefore that P_1_ and P_3_ facilitate PEXEL cleavage and thus help to release the exported proteins from the ER membrane where they are originally anchored following ER import.^11^ In comparison, the purpose of the last conserved P_2_′ glutamic acid/glutamine/aspartic acid (E/Q/D) residue of PEXEL is less certain. Some evidence suggests that following cleavage, the P_1_′ and P_2_′ residues, which now cap the mature PEXEL protein, play a role in promoting export across the PVM.^1^, ^14^, ^34^ After cleavage, the exposed xE/Q/D motif becomes N‐terminally acetylated^35^ resulting in an Ac‐ xE/Q/D cap that was thought to serve as a ‘barcode’ or a recognition motif for HSP101 engagement in the PV.^36^ Contrary to this, however, other studies have found that the P_2_′ amino acid also influences the efficiency of PEXEL processing or does not influence export at all.^37^, ^38^, ^39^

Here, we present evidence that the P_2_′ residue has a dual function. For some PEXEL proteins, P_2_′ mutations greatly reduce PMV cleavage increasing protein retention in the ER and reducing export. For other proteins, P_2_′ mutations do not inhibit PMV cleavage as much and PEXEL proteins can reach the PV but are translocated less efficiently. We also provide evidence that the length of the region downstream of the PEXEL motif regulates the degree of cargo interaction with HSP101 and ultimately affects protein export across the PVM.

## 2 RESULTS

### 2.1 A systematic review of PEXEL motif mutants reveals that a single point mutation of P_2_ ′ into a positively charged residue produces the most consistent export‐blocking effect

To better understand the contribution of the PEXEL P_2_′ position on protein export, we conducted a review of P_2_′ mutagenesis experiments that have been performed on various PEXEL proteins (Table S1). We sorted the mutants based on the amino acids incorporated into the P_2_′ position and linked this to the export phenotype, PEXEL processing pattern and N‐terminal acetylation (Table S1 ‘PMV cleavage’ and ‘N‐acetylation’) reported for the corresponding mutant. This in general revealed that alanine (A) mutations in the P_2_′ position have a limited or uncertain effect on export (Table S1). For example, Tarr et al. reported that when the P_2_′ position of KAHRP (knob‐associated histidine‐rich protein, PF3D7_0202000) was changed to an A, the protein was exported into the RBC.^37^ This was contrary to a previous study^14^ where the reporter was mostly trapped in the PV, despite both groups using very similar constructs (i.e., the first 69aa of KAHRP leader sequence fused to GFP). The same discrepancy was also observed with P_2_′ A mutations of STEVOR and GBP130 (PF3D7_1016300) (Table S1).^1^, ^14^, ^34^, ^39^, ^40^ These apparently contradictory findings may be attributable to variations in the age of the parasites being studied and consequent differences in promoter activity, as well as to differences in the module(s) appended downstream of the PEXEL motif.^37^, ^39^ P_2_′ A mutagenesis studies of other PEXEL proteins, such as RESA (PF3D7_0102200), *Pf*EMP3 (PF3D7_0201900), REX3 (PF3D7_0936300) and murine *P. berghei* CP1 (PBANKA_1246500), did not result in any export defect^4^, ^37^, ^38^ (Table S1).

Mutation of the P_2_′ PEXEL position to positively charged amino acids appears to produce the most robust export‐blocking effect on exported proteins tested in two different studies (Table S1).^37^, ^40^ Mutation to basic amino acids would usually reverse the charge of the P_2_′ position, given that the two most common P_2_′ residues are glutamic and aspartic acids. Curiously, the P_2_′ R mutant of REX3 (PF3D7_0936300) PEXEL also moderately inhibited PMV cleavage of the motif,^37^ suggesting that P_2_′ may be involved in PEXEL processing, albeit to a lesser extent than P_1_ and P_3_.

### 2.2 P_2_ ′ lysine mutations reduce export efficiency in multiple PEXEL proteins by inhibiting plasmepsin V cleavage

**FIGURE 1:**
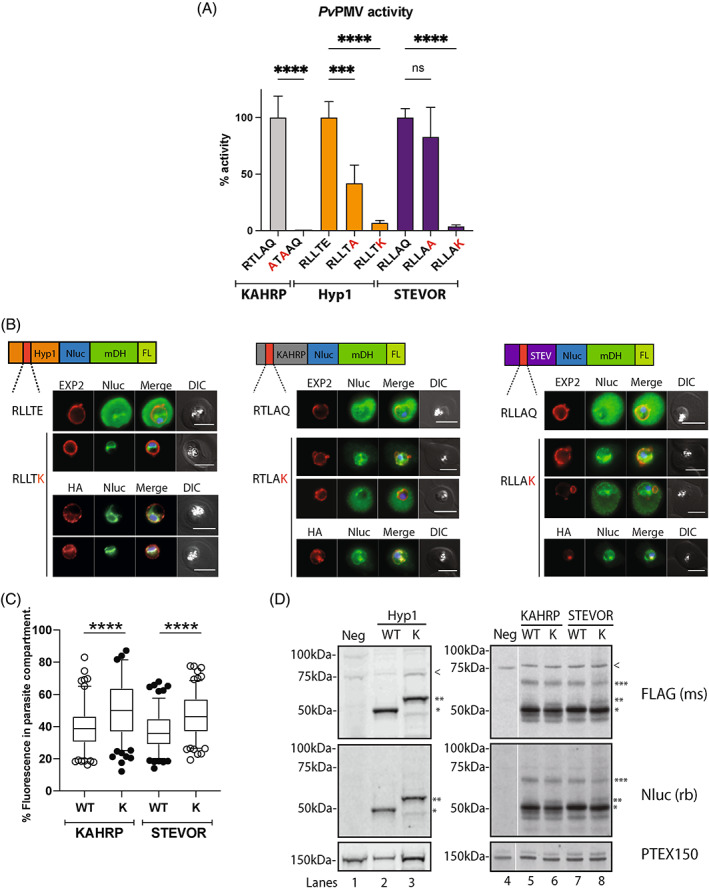
P_2_′ Alanine and Lysine mutations reduce the proteolytic processing of the PEXEL motif. (A) 5 μM fluorogenic peptides were incubated with 2 nM recombinant *Pv*PMV and assayed at 20°C. Fluorescence data was normalised to the WT substrates (*n* = 3). PEXEL motifs are indicated below the *x*‐axis with mutated resides indicated in red. Statistical significance was determined using ordinary one‐way ANOVA with Brown‐Forsythe test followed by Šídàk's multiple comparisons test. (****, *p*‐value<0.0001; ***, *p*‐value<0.001). (B) Representative IFA images (*n* = 3 independent replicates) of HSP101‐HA*glmS* parasites expressing WT and P_2_′ K PEXEL motifs of Hyp1, STEVOR, and KAHRP‐Nluc‐mDH‐FL reporter proteins. Parasite cells were probed with anti‐EXP2 and anti‐HA antibodies to visualise the PTEX components within the cell. The Nluc‐mDH‐FL proteins were localised using anti‐Nluc antibody. The red bar in the schematic picture of the construct indicates the location of the PEXEL motif. Scale bars, 5 μm. DIC, Differential Interference Contrast. DAPI (4′,6‐diamidino‐2‐phenylindole; blue) was used to stain parasite nuclei. (C) Quantification of the Nluc fluorescence signal of STEVOR and KAHRP‐Nluc‐mDH‐FL constructs within the boundary of the EXP2 staining (including the parasite cell and its PV). Quantification was performed using pooled images from three independent experiments. The following numbers of cells were used for the analysis: KAHRP P_2_′ WT (*n* = 151) and K (*n* = 161), STEVOR P_2_′ WT (*n* = 135) and K (*n* = 145). Box and whisker plot represents 25th–75th and 5th–95th percentiles, respectively. Statistical significance was determined using ordinary one‐way ANOVA. (****, *p*‐value<0.0001). (D) Representative western blot (*n* = 3) of lysates made from mid‐stage trophozoites expressing Nluc‐mDH‐FL constructs probed with anti‐FLAG and anti‐Nluc IgGs. The identity of various protein species was based on their observed sizes. ***, full length proteins. ←, **, mis‐cleaved P2’ K Hyp1 (58.1 ± 1.2 kDa, n = 10), KAHRP (51.4 ± 1.2 kDa, n = 3) and STEVOR (53.8 ± 1.7 kDa, n = 3)‐Nluc‐mDH‐FL. *, correctly PEXEL‐cleaved species of the Nluc‐mDH‐FL proteins. < is a cross‐reactive protein. Hyp1‐Nluc‐mDH‐FL blots are shown separate to KAHRP and STEVOR blots as they were run on different gels. The Neg lane contains protein from parasites not transfected with the Nluc‐mDH‐FL reporter and were part of the same blot as the KAHRP and STEVOR samples but on non‐adjacent lanes indicated by the white vertical line. Blots were probed with anti‐PTEX150 antibody as a loading control.

To clarify the effects of P_2_′ mutations of different PEXEL proteins expressed under the same conditions we synthesised several fluorogenic peptides containing wildtype PEXEL motifs and mutations thereof of three different PEXEL proteins and determined how well they were cleaved by recombinantly‐expressed *P. vivax* plasmepsin V (*Pv*PMV) (Table S2).^41^ *Pv*PMV was employed as this protease was more experimentally amenable than the *P. falciparum* equivalent.^41^, ^42^ A peptide containing the KAHRP (PF3D7_0202000) PEXEL sequence (RTLAQ) with P_3_ R to A and P_1_ L to A mutations (ATAAQ) served as a control and was not efficiently cleaved by *Pv*PMV compared to the wildtype sequence (Figure 1A). We then introduced P_2_′ mutations into the PEXEL motifs of Hyp1 (PF3D7_0113300) and STEVOR (subtelomeric variable open reading frame, PF3D7_0200400) (RLLTE and RLLAQ, respectively), changing the P_2_′ residue from E to lysine (K) for Hyp1 and from Q to K for STEVOR. In both cases, the P_2_′ K fluorogenic peptides were not efficiently cleaved indicating that P_2_′ K strongly inhibits PEXEL processing (Figure 1A).

Previously, a GFP‐tagged STEVOR with P_2_′ A mutation was shown to be trapped at the parasite periphery by microscopy.^34^ However, the PEXEL processing status of this mutant was not investigated. We therefore included P_2_′ A mutants of both Hyp1 and STEVOR in our cleavage assay. Compared to the wildtype peptides, cleavage of the P_2_′ A mutants of Hyp1 and STEVOR was moderately inhibited, but this was only statistically significant for Hyp1 (58%). The P_2_′ A mutant is thus less efficiently cleaved than WT and but more efficiently cleaved than the charge reversal P_2_′ K mutant (Figure 1A).

We next sought to determine how the P_2_′ K mutations would affect the trafficking and proteolytic processing of these proteins in parasite‐infected RBCs. To generate reporters specific for these proteins we fused the first 113aa of Hyp1, containing the wildtype PEXEL motif RLLTE or a P_2_′ K mutant version, to a reporter cassette comprising nanoluciferase (Nluc), murine dihydrofolate reductase (mDH) and three FLAG epitopes (FL).^42^ For KAHRP, we fused the first 105aa, including the wildtype PEXEL motif RTLAQ or a P_2_′ K mutant version, to Nluc‐mDH‐FL. For STEVOR, we fused the first 99aa, including the wildtype PEXEL motif RLLAQ or the corresponding P_2_′ K mutant version, to the same reporter. All six constructs were transfected into the HSP101‐HA*glmS* or the HSP101‐HA parasite background line to enable direct comparison between the wildtype and P_2_′ K mutants. Although microscopy and western blot data of wildtype and P_2_′ K Hyp1‐Nluc‐mDH‐FL have been reported previously^42^ we have presented new images and blots here for comparison to KAHRP and STEVOR. All P_2_′ wildtype (P_2_′ WT) reporters were exported with most of the signal in the RBC compartment (Figures 1B, C, S1, and S2). For the P_2_′ K mutants, Hyp1 differed from STEVOR and KAHRP with Hyp1 being largely trapped around the nucleus in the ER and P_2_′ K STEVOR and P_2_′ K KAHRP being trapped in the ER and around the parasite circumference in the PV (Figures 1B, S1, and S2). As efficient protein export was observed for some P_2_′ K KAHRP and STEVOR reporter parasites (Figures S1 and S2, respectively), the percentage of reporter signal inside the parasite compartment (including the PV) compared to the whole infected RBC was quantified. This indicated that although the efficiency of export varied widely, there was generally more reporter within the parasite compartment for the P_2_′ K reporters than for the P_2_′ WT reporters (Figure 1C).

We have previously shown that the Hyp1 P_2_′ K reporter was not efficiently cleaved at the PEXEL motif by *Pv*PMV and that this could be why this reporter was not exported and remained trapped in the ER.^42^ To determine if incorrect cleavage of the KAHRP and STEVOR reporters could also be responsible for the increased trapping of the reporters in the parasite we performed western blot analysis on parasite lysates expressing WT and P_2_′ K Hyp1‐, STEVOR‐ and KAHRP‐Nluc‐mDH‐FL constructs. In the western blot analysis of Hyp1 parasite lysates probed with anti‐FLAG and anti‐Nluc IgGs, the predominant correctly cleaved species of WT Hyp1 migrates at 50 kDa with the full‐length pre‐processed species, expected at 70 kDa, below detection (Figure 1D, lane 2, single asterisk). In contrast, cleavage of the Hyp1 P_2_′ K reporter appears to be upstream of the PEXEL motif, probably near the transmembrane domain (Figure 1D, lane 3, double asterisk).^42^ Incorrect cleavage correlates with the high ER retention observed by immunofluorescence analysis (IFA), although the mechanism for this is unknown (Figure 1B, D).^42^

In contrast, P_2_′ K STEVOR and KAHRP constructs migrated predominantly at the same size as their WT counterparts on a western blot (Figure 1D, lanes 5–8, single asterisk), indicating that the P_2_′ K STEVOR and KAHRP reporters are mostly processed within their PEXEL motif which would explain why they visually appear more efficiently trafficked to the PV and RBC relative to Hyp1 P_2_′ K (Figure 1B). However, we noted additional low abundance mis‐cleaved bands for the KAHRP and STEVOR P_2_′ K reporters that were approximately 3–4 kDa bigger than the PEXEL cleaved species (Figure 1D, lanes 6 and 8, double asterisks). We were able to detect these mis‐cleaved species using anti‐FLAG antibody, suggesting they are not C‐terminally truncated forms of the full‐length proteins (Figure 1D, Table S3) and may represent aberrant N‐terminally processed forms of the P_2_′ K KAHRP and STEVOR reporters arising from less efficient PEXEL processing, which may account for the small reduction in export. We could also detect the pre‐processed form for KAHRP and STEVOR (Figure 1D, triple asterisk). Collectively, both western blot and biochemical analyses suggest that a P_2_′ mutation, particularly to a positively charged residue, can reduce efficient cleavage and cause ER retention for Hyp1 but not for KAHRP and STEVOR. For the latter two proteins, cleavage of the P_2_′ K PEXEL is much more efficient and the proteins traffic to the PV but are translocated less effectively into the RBC than WT reporters.

### 2.3 P_2_ ′ K STEVOR and KAHRP are more soluble than P_2_ ′ K Hyp1

**FIGURE 2:**
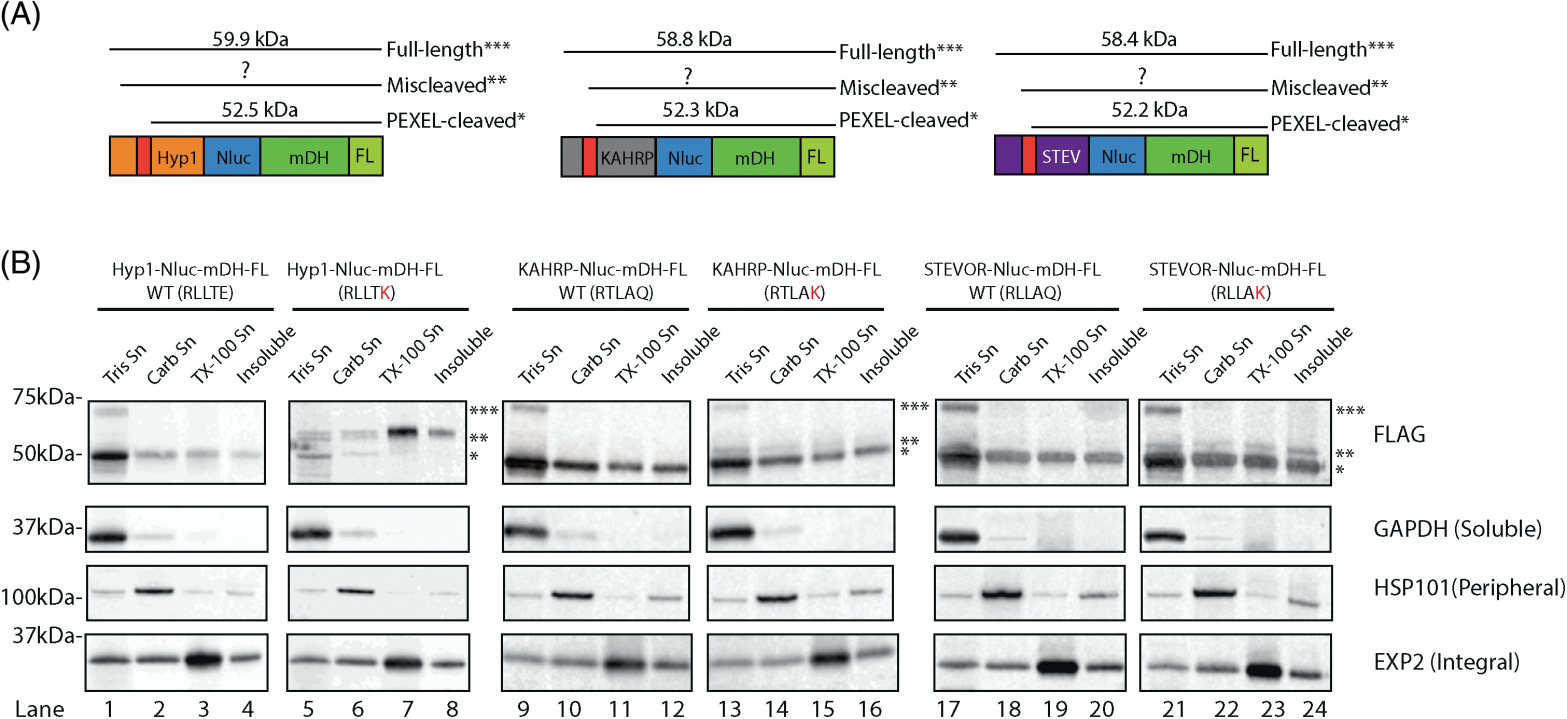
Solubility profile of Nluc‐mDH‐FL proteins. (A) Predicted full‐length and correctly processed sizes of Hyp1, KAHRP and STEVOR‐Nluc‐mDH‐FL reporter proteins. The cleavage site of the mis‐cleaved reporters is not known and so their sizes cannot be predicted as represented by question marks. (B) Western blot analysis of infected RBCs (mid‐stage trophozoites) sequentially extracted with 5 mM Tris‐Cl pH 8.0 (Tris Sn), 0.1 M Na_2_CO_3_ pH 11.5 (Carb Sn) and 1% Triton X‐100 buffer (TX‐100 Sn) to partition proteins based on their association with cellular membranes. Insoluble fraction represents the final pellet obtained after Triton X‐100 extraction. GAPDH, HSP101 and EXP2 were used as a control for the release of soluble, peripheral and integral protein, respectively. *, PEXEL‐cleaved species. **, mis‐cleaved species. ***, full‐length protein.

Consistent with our earlier findings,^42^ we observed that the ER‐trapped mis‐cleaved Hyp1 P_2_′ K reporter was poorly soluble, whereas the low‐abundance correctly processed Hyp1 P_2_′ K reporter was readily extractable by hypotonic lysis (Figure 2B, lanes 5–8). The poor solubility of the mis‐cleaved species may partly account for its ER retention. To determine if PV trapping of STEVOR and KAHRP P_2_′ K reporter proteins was likewise due to reduced solubility, we performed protein solubility assays on parasite lines expressing the WT and P_2_′ K Nluc‐mDH‐FL reporters (Figure 2A). The correctly cleaved forms of P_2_′ K KAHRP‐ (Figure 2B, lanes 13–16, single asterisk) and STEVOR‐Nluc‐mDH‐FL (Figure 2B, lanes 21–24, single asterisk) were largely found in the soluble fraction (Tris Sn), although some was present in the other fractions as well. Additionally, we noticed that the putative low‐abundance higher molecular weight forms of P_2_′ K KAHRP and STEVOR (Figure 1D) were again present and evenly distributed in all fractions, including the soluble fraction (Figure 2B, lanes 13–16 and 21–24, double asterisks). These data suggest that mis‐cleaved P_2_′ K reporters may remain trapped in the ER because they are less soluble than the correctly cleaved reporters, although the mechanism behind this is not obvious since both the size of the mis‐cleaved P_2_′ K proteins and previous mass spectrometric analysis suggest they lack their hydrophobic signal peptides.^42^ When correctly processed, however, the P_2_′ K reporters are more soluble, which may facilitate their traffic beyond the ER to at least the PV.

### 2.4 The length of the spacer region is essential for protein translocation across the parasitophorous vacuole membrane

**FIGURE 3:**
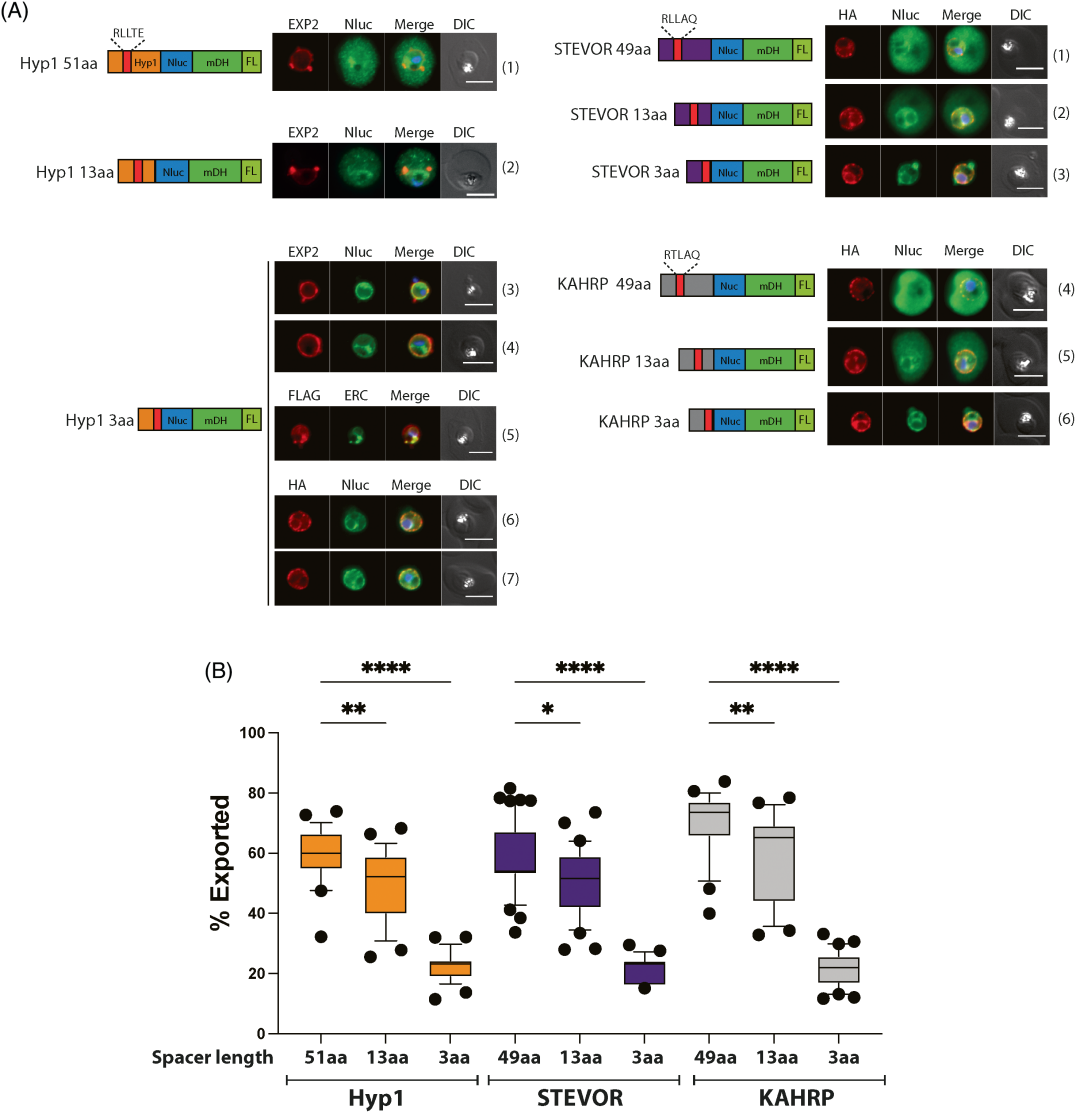
Sequential truncation of the spacer region caused trapping of the Nluc‐mDH‐FL constructs in the ER and PV of the parasite. (A) Representative IFA images (*n* = 3 independent replicates) of HSP101‐HAglmS parasites expressing Hyp1/STEVOR/KAHRP‐Nluc‐mDH‐FL proteins with various spacer lengths (see diagrams). Parasite cells were probed with anti‐EXP2 and anti‐HA antibodies to visualise the PTEX components within the cell. Anti‐*Pf*ERC antibody was used to visualise parasite's ER. The Nluc‐mDH‐FL proteins were either probed with anti‐Nluc or anti‐FLAG antibodies. Scale bars, 5 μm. DIC, Differential Interference Contrast. DAPI (4′,6‐diamidino‐2‐phenylindole; blue) was used to stain parasite nuclei. (B) Quantification of export of the spacer truncation Nluc‐mDH‐FL constructs presented in (A). The following numbers of cells were used for the analysis. Hyp1 constructs: *n* = 25 (51aa), 25 (13aa), 28 (3aa) cells; STEVOR constructs: *n* = 53 (49aa), 32 (13aa), 23 (13aa) cells; KAHRP constructs: *n* = 26 (49aa), 23 (13aa), 30 (3aa) cells. Box and whisker plot represents 25th–75th and 10th–90th percentiles, respectively. Statistical significance was determined using one‐way ANOVA with Brown‐Forsythe test followed by Tukey’s multiple comparisons test. ****, *p*‐value<0.0001; **, *p*‐value<0.01; *, *p*‐value = 0.0159).

Thus far our data have indicated that the amino acid at the P_2_′ position is important for the correct cleavage of Hyp1 but less so for KAHRP and STEVOR indicating that other residues within and/or bordering the PEXEL motif may also be important for accurate cleavage. Earlier work has shown that truncation of the amino acid sequence (termed spacer region) that separates the PEXEL motif from a downstream folded protein, such as GFP, influences export.^22^, ^24^ Interestingly, the N‐terminal regions of PNEPs are functionally exchangeable with this spacer region of PEXEL proteins^39^ and replacement of the spacer region with the N‐terminal sequence of a PV‐resident protein inhibits export,^4^ suggesting that this region may comprise a bona fide export signal. To investigate whether the spacer region has a role in binding to PTEX, the spacer region of the Hyp1‐, STEVOR‐ and KAHRP‐Nluc‐mDH‐FL constructs were C‐terminally truncated, from their original lengths of ~50aa, down to 13aa and 3aa preceding the folded domain of Nluc (Figure 3A, B). IFAs of trophozoite‐infected RBCs expressing the truncation constructs showed reduced export with reduced spacer length in all three constructs. Quantification of the exported signal across the cell population further revealed that truncation from ~50aa to 13aa reduces export by ~10%–20%, while export was strongly reduced in 3aa spacer constructs, showing a marked ~80% reduction in fluorescence signal relative to the control (Figure 3A–C). This observation contrasts with the mutations of the P_2_′ PEXEL motif alone, performed in the previous section (Figure 1), and other studies, which displayed variable export‐blocking phenotypes with different PEXEL protein sequences.^1^, ^14^, ^34^, ^37^ Co‐labelling of microscopy images with EXP2 (PV marker) and *Pf*ERC (ER marker) further indicated that Hyp1‐Nluc‐mDH‐FL with a 3aa spacer accumulated mainly in the PV with some signal in the ER overlapping with HSP101‐HA (Figure 3A panels 3–7).

IFAs using anti‐Nluc with STEVOR‐Nluc‐mDH‐FL and KAHRP‐Nluc‐mDH‐FL parasites showed that the 13aa and 3aa spacer reporters behaved similarly to the Hyp1‐reporter (Figure 3B, panels 2, 3 and 5, 6) and also displayed the highest co‐localisation with the HA‐tagged translocon component HSP101, which we have shown resides within the ER in addition to the PV.^42^, ^43^

**FIGURE 4:**
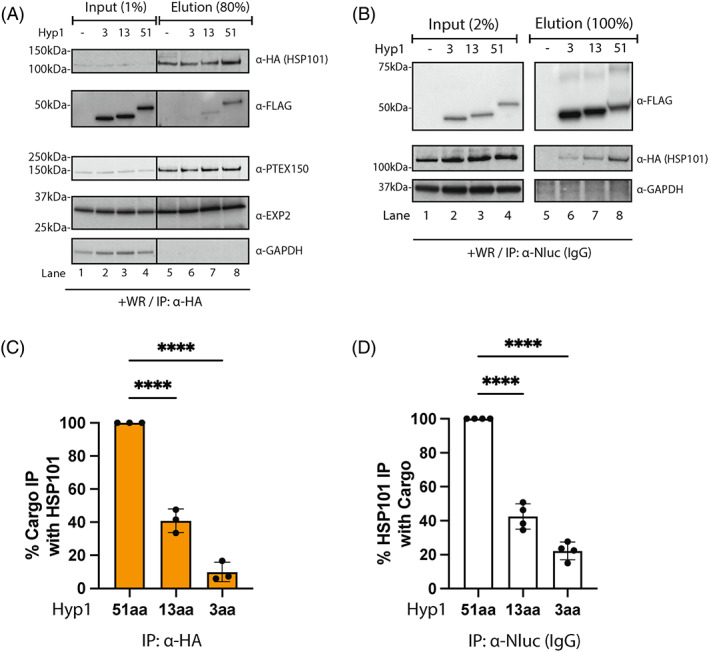
Truncation of the spacer region of Hyp1‐Nluc‐mDH‐FL reduces its interaction with HSP101. (A) Western blot of anti‐HA immunoprecipitation (IP) of HSP101‐HA*glmS* parasites expressing various truncations of the Hyp1 spacer region (*n* = 3) as indicated above each lane. HSP101‐HA*glmS* parasites not transfected with a Hyp1‐Nluc‐mDH‐FL reporter are represented by ‘‐’. Immunoblots were performed to detect other PTEX components (EXP2 and PTEX150) as a positive control and GAPDH as a negative control. Hyp1 spacer mutants were visualised using anti‐FLAG antibody. (B) Western blot of the reciprocal anti‐Nluc IP of the truncated spacer reporters (*n* = 4). Immunoblots were performed using anti‐HA to detect HSP101 and GAPDH as a negative control. (C) and (D) Densitometry of the IP performed on (A; left) and (B; right). For anti‐HA IP, the intensities of co‐immunoprecipitated 13aa and 3aa spacer Hyp1‐Nluc‐mDH‐FL were normalised to the value of the 51aa spacer. For anti‐Nluc IP, the intensities of co‐immunoprecipitated HSP101 bands in the 13aa and 3aa spacers were normalised to the 51aa spacer. Error bars, ±SD. Statistical significance was determined using one‐way ANOVA with Brown‐Forsythe test followed by Tukey’s multiple comparisons test. (****, *p*‐value<0.0001).

Truncation of the spacer did not appear to reduce processing of the PEXEL motif in this context as western blots of the Hyp1‐Nluc‐mDH‐FL truncation constructs showed that each reporter protein migrated according to a predicted mass consistent with PMV‐processed versions of the proteins (Figure 4A, B, lanes 2–4, Table S4). Taken together, these results show that the length of the spacer is important for export, post‐PEXEL processing.

### 2.5 Truncation of the spacer region reduces cargo binding with HSP101

The observed co‐localisation of all spacer constructs with the translocon components EXP2 and HSP101 in the PV was perplexing as the truncated proteins appeared to have processed PEXEL N‐termini but were unable to be exported, suggesting a failure to correctly engage with PTEX. We therefore sought to determine if the truncated PEXEL proteins could bind HSP101 by co‐immunoprecipitation. To do this, the Hyp1 truncation constructs (51aa, 13aa and 3aa Hyp1‐Nluc‐mDH‐FL) were transfected into the HSP101‐HA*glmS* parasite line.^42^ These parasites were grown to the ring stage and treated with WR99210 for 24 h to stabilise the folding of the murine dihydrofolate reductase, that had previously been demonstrated to stall the cargo unfolding process within PTEX, thereby trapping and stabilising the cargo's interaction with PTEX.^16^, ^43^, ^44^, ^45^ The whole trophozoite‐infected RBCs were lysed and incubated with anti‐HA‐IgG agarose to immunoprecipitate the HA‐tagged HSP101 from the sample. Western blot analysis of the eluates revealed a significantly reduced amount of Hyp1‐Nluc‐mDH‐FL co‐eluted with HSP101 with decreasing length of the spacer, with 60% and 90% reduction (*n* = 3) observed in the 13aa and 3aa spacer, respectively, relative to the 51aa spacer Hyp1‐Nluc‐mDH‐FL (Figure 4A, lanes 6–8 and 4C). Importantly, the experiment was performed in the presence of a stabilising ligand WR99210, suggesting that the 13aa and 3aa spacer truncation mutants did not proceed to the unfolding step within PTEX.

The same samples were also subjected to a reciprocal co‐immunoprecipitation using anti‐Nluc antibodies to pull down the Hyp1‐Nluc‐mDH‐FL truncation proteins and we consistently saw a gradual reduction in the amount of HSP101 co‐eluted as the length of the spacer was shortened (Figure 4B, lanes 6–8 and 4D). We plotted the normalised % exported as observed by IFA and the amount of cargo (Hyp1‐Nluc‐mDH‐FL) bound with HSP101 and we saw a remarkable correlation between these two variables, suggesting that the level of cargo binding with HSP101 determines its exportability (compare Figure 3C with Figure 4B, D). Taken together, these results demonstrated that the spacer region regulates cargo engagement with PTEX, particularly with HSP101 which is possibly the first point of contact cargo has with PTEX.^19^, ^46^

### 2.6 The sequence requirements of the 13aa spacer for export are relatively unconstrained

**FIGURE 5:**
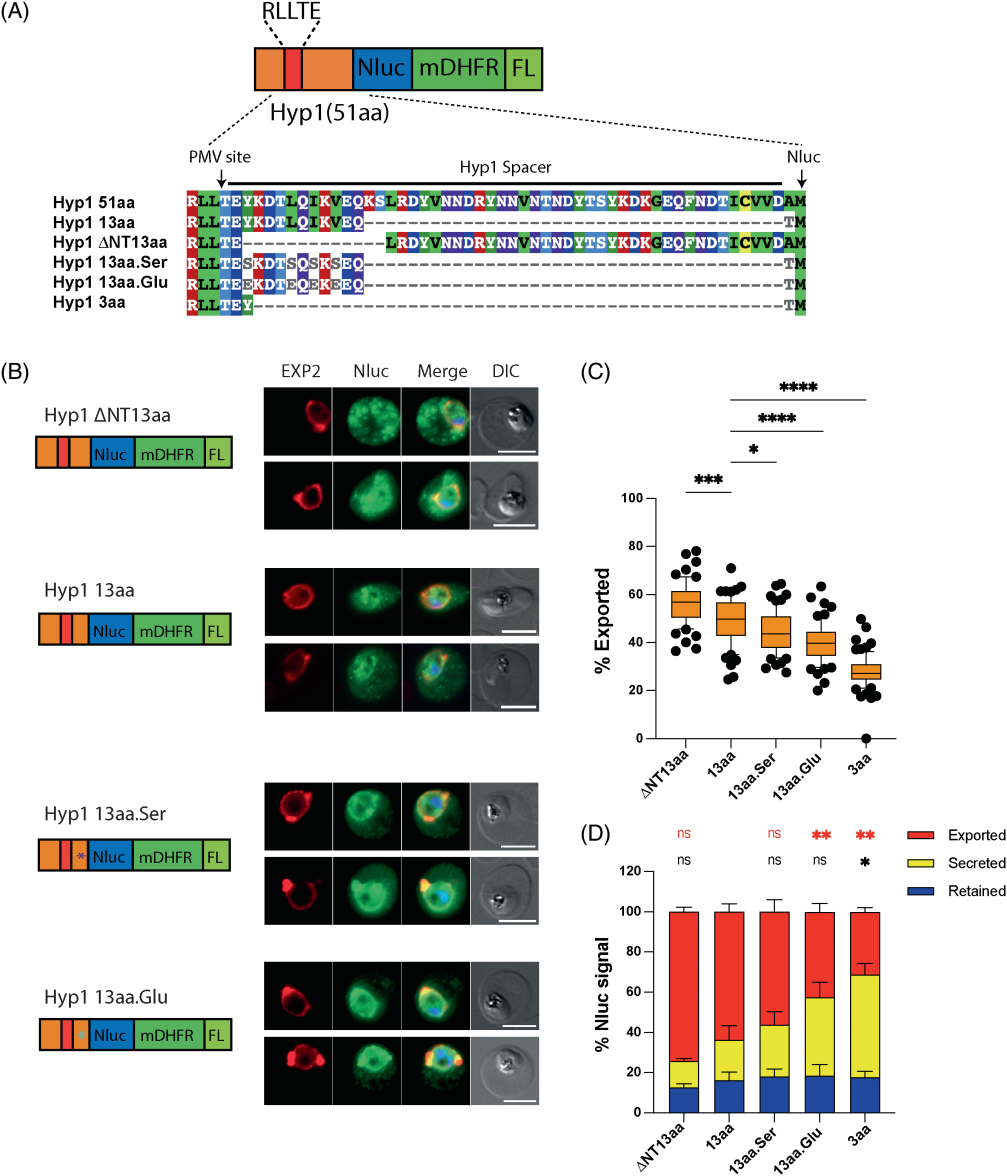
The sequence requirements of the 13aa spacer for export are relatively unconstrained. (A) Alignment of the sequences of Hyp1 spacers (including the PEXEL motif) whose trafficking was investigated. (B) Fluorescence microscopy of Hyp1 spacers detected with rabbit anti‐Nluc antibody, using mouse anti‐EXP2 as a PVM marker, indicated that the ∆NT13aa, 13aa and 13aa.Ser reporters were exported efficiently whereas the Hyp1 13aa.Glu reporter appeared to be more strongly retained in the parasite. (C) Quantification of the % of exported protein signal beyond the EXP2 boundary into the infected RBC compartment indicated that the degree of export was progressively reduced in the Hyp1 13aa.Ser, Hyp1 13aa.Glu and Hyp1 3aa parasites relative to the Hyp1 13aa parasites. The Hyp1 ∆NT13aa parasites exported more efficiently than the Hyp1 13aa parasites. Quantification of export was performed using pooled images from three independent experiments. The following numbers of cells were used for the analysis: 64 (∆NT13aa), 60 (13aa), 64 (13aa.Ser), 64 (13aa.Glu) and 77 (3aa). Box and whisker plot represents 25th–75th and 10th–90th percentiles, respectively. Statistical significance was determined using one‐way ANOVA with Brown‐Forsythe test followed by Dunnett’s multiple comparisons test for comparison to the 13aa reporter. (*, *p*‐value, <0.05). (D) Nanoluciferase export assay with the same parasite lines produced a similar trend to the microscopic imaging but specifically indicated that most of the reduction in export in the mutant Hyp1 13aa and Hyp1 3aa spacers was due to retention in the PV rather than the parasite. Statistical significance was determined using one‐way ANOVA with Brown‐Forsythe test followed by Dunnett's multiple comparisons test for comparison to the 13aa reporter. (*, *p*‐value <0.05, **, *p*‐value <0.01).

Having observed that the 13aa Hyp1 spacer still permitted a reasonable amount of export compared to the full‐size 51aa spacer we decided to use this as a basis for subsequent mutagenesis experiments due to its small size. The spacers of PEXEL proteins appear to possess little obvious sequence information for putative PTEX recognition apart from appearing relatively unstructured with no conserved domains. To assess whether the 13aa spacer may contain some cryptic trafficking information, we decided to remove it from the original 51aa spacer and fuse this new ∆NT13aa spacer to the Nluc‐mDH‐FL reporter and express this in parasites (Figure 5A). Western blots of the Hyp1 ∆NT13aa‐Nluc‐mDH‐FL parasites indicated the reporter protein was of a size consistent with correct PMV cleavage (Figure S3). Microscopy of the ∆NT13aa reporter showed it was efficiently exported and quantification of the fluorescence intensity implies a higher degree of export of the ∆NT13aa reporter than the 13aa spacer (Figure 5B, C). This result indicated that the ∆NT13aa spacer was competent for export and that the information contained in the 13aa spacer is not essential for export of the Hyp1‐Nluc‐mDH‐FL reporter.

Next, we investigated whether certain amino acids within the 13aa spacer were necessary for export. As protein chaperones tend to recognise misfolded proteins via their hydrophobic residues,^47^ the four hydrophobic amino acids tyrosine (Y), L, isoleucine (I), and valine (V) in the 13aa Hyp1 spacer TEYKDTLQIKVEQ were mutated to determine if this reduced export. In the absence of predictive tools for HSP100 chaperones, we used a binding predictor for the ER HSP70 chaperone BIP^47^ that scans 7aa peptide windows and found that mutation of the hydrophobic amino acids to the polar amino acid serine, produced lower scores than for other amino acids (Figures 5A and S4A–C). Therefore, we replaced the hydrophobic amino acids in the 13aa spacer with serine and transfected this Hyp1 13aa.Ser reporter into parasites where, by western blot, the Hyp1 13aa.Ser reporter was of a size consistent with correct PMV cleavage (Figure S3). Microscopic analysis of the Hyp1 13aa.Ser parasite‐infected RBCs indicated that this mutant was exported less efficiently than the WT 13aa spacer (Figure 5A–C).

Next, the hydrophobic amino acids of the 13aa spacer were mutated to glutamic acids (Hyp1 13aa.Glu) as these mutations were scored most poorly by the BIP binding predictor (Figure S4A–C). Western blots of Hyp1 13aa.Glu‐Nluc‐mDH‐FL parasites indicated PMV processing was correct and microscopic analysis of parasite‐infected RBCs expressing the reporter indicated the Hyp1 13aa.Glu protein was exported significantly less well than the Hyp1 13aa reporter (Figure 5B, C). These results indicated that although there is probably a great deal of flexibility in the amino acid sequences of spacers, some attributes are required such as the presence of hydrophobic amino acids that could be important for general chaperone binding.

As it can be difficult to visibly discern cargo trapped in the PV versus that inside the parasite we employed a recently‐developed protein export assay based on the release and detection of Nluc bioluminescence from differentially lysed cellular compartments.^43^, ^48^ We saw that the Nluc bioluminescence signal exported into the RBC compartments closely followed the same trend as the spacer reporter parasites whose export was measured visibly by microscopy (compare Figure 5C, D). However, the % exported in the Nluc bioluminescence export assay was however often 10%–20% greater than the microscopy measurement, probably because bioluminescence is highly sensitive and can detect lower protein levels dispersed in the large RBC compartment (Figure 5D). Additionally, microscopy does not measure RBC signal in front of or behind the parasite which may result in underestimation of the total signal. Interestingly, the bioluminescence signal of the reporters secreted into the PV compartment increased as export into the RBC compartment decreased indicating that as the spacer length shrank, or its hydrophobic residues were mutated, the proteins were still able to traverse the parasite plasma membrane but were less efficiently translocated into the RBC by PTEX, leaving them to accumulate in the PV. It is also worth noting that the signal retained in the parasite was relatively constant indicating spacer length or mutations did not reduce secretion into the PV.

## 3 DISCUSSION

Here, we sought to understand what element(s) of PEXEL proteins govern recruitment of PTEX to facilitate the export of proteins into the RBC compartment. It was previously hypothesised that the last conserved residue of the PEXEL, which remains on the mature protein, is responsible for interacting with PTEX.^1^, ^2^, ^14^ Our data partly support this in that mutation of the P_2_′ residue to K can lead to increased trapping of some PEXEL proteins in the PV, most notably for the KAHRP and STEVOR reporters. The P_2_′ K mutation can also greatly reduce the efficiency of PMV cleavage and as such increase protein trapping in the ER, as shown for the Hyp1 reporter. Apart from the efficiency of cleavage of the PEXEL motif for governing PTEX's interaction with the cargo proteins, the spacer region downstream of the PEXEL that separates the PEXEL from the folded functional Nluc region of the reporter protein is also important for trafficking. As the length of spacer increased, so did the reporter's binding to PTEX and the efficiency with which it was exported into the RBC compartment. A small spacer length of 13aa was found to still result in efficient export with hydrophobic residues in the spacer important for export efficiency. Reporters with short or mutated spacers appeared to be still efficiently cleaved by PMV but interacted weakly with PTEX, leaving them trapped in the PV compartment and unable to be exported. One caveat of this study is that our reporter had a folded Nluc domain a defined distance from the upstream PEXEL cleavage site, and we are unsure if similar rules will generally apply to native PEXEL proteins, many of whose structures and functions are unknown.

Other studies have shown that the amino acid in the last position of export motifs is important for export. For example, reporter proteins containing the oomycete effector motif RxLR,^30^, ^39^ which lack the P_2_′ residue, were not cleaved by PMV and failed to promote export into the host cell.^30^, ^39^ Of all possible mutations of P_2_′, only charge reversal P_2_′ mutations, either to R or K, strongly inhibit PMV cleavage^37^ whilst a single A mutation was observed to cause a variable export phenotype.^4^, ^37^ This was evident from the small reduction of cleavage observed in the in vitro cleavage assay with P_2_′ A mutation compared to the K mutation. Despite this, the strong inhibition of in vitro cleavage of STEVOR P_2_′ K did not translate to what was observed in parasites as the P_2_′ K reporter protein was efficiently cleaved. It seems likely that suboptimal amino acid variants at PEXEL P_2_′ are still permissive for PMV cleaving in the cellular context, possibly because of the fast kinetics of the proteolytic reaction in vivo or due to concentrated substrate in the ER that permits cleavage of substrates with suboptimal cleavage sites. It is also possible that *Pf*PMV in parasites is more active against the P_2_′ mutants than the *Pv*PMV used in the peptide cleavage assay.^49^, ^50^

Having previously observed that the ER‐trapped Hyp1 P_2_′ K mutant, which was mis‐cleaved by an unknown protease upstream of the PEXEL, became quite insoluble and remained in the ER,^42^ we explored how the solubility of cleaved PEXEL reporters can influence their export. In contrast to the mis‐cleaved Hyp1 P_2_′ K reporter,^42^ the solubility of KAHRP and STEVOR P_2_′ K reporter proteins did not appear to decrease relative to their WT counterpart. Given that the KAHRP and STEVOR mutants (a) were efficiently cleaved and trafficked to the PV and (b) did not show a marked decrease in solubility, the reduction in export of these mutants relative to their WT counterparts, albeit slight, is curious. It is plausible that the P_2_′ residue of these proteins is not important for the initial interaction with HSP101 but rather for commitment to stronger downstream interactions with the whole of PTEX required for cargo unfolding and export. Further investigation will be required to substantiate this hypothesis.

Our results also argue that PTEX, more specifically HSP101, recognises a wider region in the cargo than the PEXEL motif and that this is important for export. Truncation of the spacer region in the three different reporter constructs used in this study consistently blocked export without apparently affecting PEXEL processing. Microscopic analysis of the 3aa spacer Nluc‐mDH‐FL constructs clearly showed that the protein is trapped within the PV area where PTEX is located, consistent with previous reports.^4^, ^24^ Despite this co‐localisation, we have shown that spacer mutants that bind less strongly to HSP101 are exported less efficiently.

**FIGURE 6:**
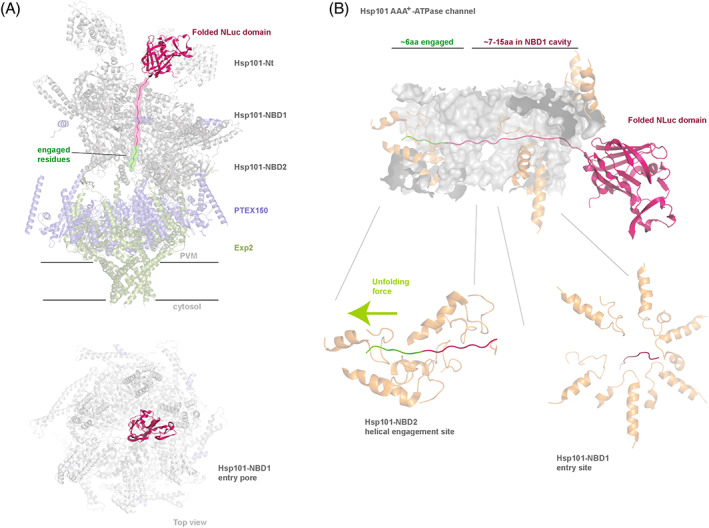
Hypothetical model for the HSP101‐cargo recognition. (A) Side and top views of a scale model of PTEX based on cryoelectron microscopy structure showing how the Hyp1spacer with attached Nluc (red) could extend into PTEX's central cavity.^19^ (B) Enlarged view of the central HSP101 cavity showing the spacer length required to engage the helical protein binding‐regions of HSP101‐nucleotide binding domain 2 (NBD2) which could apply an unfolding force to the cargo proteins for translocation. PTEX model was adapted from the published CryoEM structures (PDB: 6E10 and PDB: 6E11).^19^

While the molecular mechanism of PTEX cargo recognition remains to be elucidated, our data have shed some light into how this process may occur. We found that while a 13aa spacer was sufficient to facilitate modest export, a longer 51aa spacer promoted stronger cargo binding to HSP101, suggesting that perhaps the increased length of the unstructured N‐terminal polypeptide increases the likelihood of the cargo stably binding to HSP101. Clp/HSP100 chaperones generally require a recognition signal of at least 10–20 broadly diverse amino acids to initiate polypeptide unfolding and translocation.^51^, ^52^ Our data are consistent with this model and suggest that HSP101 also requires a region within the N‐terminal portion of the cargo protein to initiate cargo translocation. This could explain why single P_2_′ K point mutation in the mature PEXEL motif is not enough to prevent the interaction altogether and why the N‐termini of PNEPs, despite lacking a mature PEXEL motif, can still act as an export signal.^39^ Cargo recognition in AAA+ ATPases, particularly the family of Clp/HSP100 chaperones, begins with the pre‐unfolding step that is initiated by a low‐affinity probabilistic binding of the chaperone to a loosely folded or aggregated region of a protein, followed by a commitment step where the ATPase binds more stably to the cargo protein before unfolding and threading the protein through the chaperone's central cavity.^53^ It has been shown for the AAA+ ATPase ClpXP, that the length of the cargo polypeptide bound to the inner cavity of the ClpXP affects the commitment step, such that longer polypeptides seem to promote more successful commitment and subsequent unfolding.^54^, ^55^ Clp/HSP100 chaperones, particularly ClpB and HSP104, are thought to have a similar pre‐unfolding step.^52^, ^53^, ^56^ We therefore propose a model whereby a longer spacer region may increase accessible areas for the initial probabilistic binding step, or stabilise association of exported proteins to HSP101, subsequently leading to less frequent dissociation from the unfoldase (Figure 6A). Consistently, the Hyp1‐Nluc‐mDH‐FL reporter with a short 3aa spacer region exhibited low‐level affinity to HSP101 that greatly reduced export, suggesting that the cargo may have initially associated with HSP101 but later dissociated from the unfoldase because there was insufficient net affinity to proceed to the commitment step.

We also explored which amino acids of the spacer region could promote or inhibit HSP101 binding. The subunits of the second nucleotide‐binding domain (NBD2) of HSP101 contain conserved tyrosine residues which are thought to bind the unfolded cargo protein via hydrophobic interactions to help ratchet the cargo through HSP101.^19^ As the HSP101 subunits undergo allosteric changes powered by ATP hydrolysis, the tyrosines move up and down to help grip and pull on the cargo (Figure 6B). Since it is possible that the tyrosine residues interact with the hydrophobic residues in the spacer region, we mutated the four hydrophobic residues in the 13aa Hyp1 spacer and this was shown to reduce export. Export reduction was particularly strong for the mutation of hydrophobics to charged residues (E) compared to polar residues (S). The deletion of the 13aa spacer region from the 51aa acid spacer still resulted in strong export as the next 13aa downstream from the first 13aa still contained four hydrophobic residues. One possible reason why the 13aa spacer preceding the globular Nluc region was exported much better than the 3aa spacer was that the longer spacer could project further into HSP101′s central cavity, down into NBD2 where it could engage the cargo‐binding tyrosine residues (Figure 6B).

In conclusion, our data suggest dual functions for the P_2_′ position of PEXEL proteins. The first is that it forms part of the PMV recognition sequence for cleavage. In some proteins such as Hyp1, mutation of P_2_′ to K greatly reduces PMV binding and/or successful proteolytic activity but in other PEXEL proteins such as STEVOR and KAHRP P_2_′ is not as critical for PMV cleavage in the cellular context. A lack of PMV cleavage probably results in ER retention as evidenced by the substantial retention for poorly cleaved Hyp1 versus efficiently cleaved STEVOR and KAHRP. Successful cleavage of the PEXEL P_2_′ K mutant appears to permit trafficking to the PV as STEVOR and KAHRP more efficiently reach the PV than the poorly cleaved Hyp1. Once in the PV, however, the STEVOR and KAHRP P_2_′ K reporters were not exported into the RBC compartment as efficiently as their wild‐type counterparts. This could be due to the reporters not being efficiently recognised by PTEX or because the reporters were in a PTEX‐free sub‐compartment of the PV. The latter, however, is unlikely, because recent split‐GFP experiments indicate that PV‐resident proteins have full access to PTEX.^57^ It is possible therefore that the charge reversal P_2_′ mutant somehow binds to PTEX less efficiently leading to less engagement, unfolding and eventual export into the RBC compartment.

## 4 MATERIALS AND METHODS

### 4.1 Culture of *P. falciparum* transfectants

Asexual blood‐stage *P. falciparum* (3D7 background) was cultured according to the established protocol.^58^ Cultures were routinely maintained in complete RPMI media containing RPMI‐1640 base medium supplemented with 2.5 mM HEPES, 367 μM hypoxanthine, 31.25 μg/mL Gentamicin, 25 mM NaHCO_3_ and 0.5% (w/v) Albumax II (Invitrogen). Prior to transfection, 100 μg of plasmid DNA was resuspended in TE and cytomix (25 mM HEPES, 120 mM KCl, 0.15 mM CaCl_2_, 2 mM EGTA, 5 mM MgCl_2_, 10 mM K_2_HPO_4_/KH_2_PO_4_ pH 7.6) and mixed with packed RBCs as per Hasenkamp et al.^59^ After electroporation using Gene Pulser XCell System (BioRad), the uninfected RBCs were mixed with 20 μL of HSP101‐HA*glmS* trophozoite‐stage parasites^42^ which were allowed to invade the transfected RBCs for 2 cell cycles before starting selection with 2.5 μg/mL blasticidin S.

### 4.2 Generation of PEXEL P_2_ ′ mutants and spacer truncation constructs

The expression of Hyp1/ STEVOR/ KAHRP‐Nluc‐mDH‐FL reporters was driven by a bidirectional *Plasmodium berghei* EF1α promoter that also controlled expression of the blasticidin deaminase drug resistance cassette. The plasmid pEF‐Hyp1‐Nluc‐mDH‐FL was derived from plasmid pEF‐Hyp1‐Nluc‐DH‐APEX.^43^ The Hyp1 component of this plasmid contained the first 113aa of Hyp1 (PF3D7_0113300), including the RLLTE PEXEL motif.^43^ A synthetic murine dihydrofolate reductase (mDH) gene fragment with C‐terminal 3x FLAG epitopes (Bioneer Pacific) was ligated into the Nluc‐DH‐APEX plasmid using *SpeI* and *MluI* enzymes to remove the previous mDH‐APEX gene cassette.

Generation of the P_2_′ lysine (K) mutation of the Hyp1‐Nluc‐mDH‐FL was performed as follows: Hyp1 region was first amplified as two overlapping PCR fragments with the primer pairs 1 & 2 and 3 & 4 (Table S5). The overlap region between these two PCR products contained the K mutations. PCR fragments were then sewn together with primer pair 1 & 4 via overlapping PCR and ligated into the pJET1.2/blunt plasmid (ThermoFisher Scientific). Mutagenesis was confirmed via standard Sanger sequencing (service provided by Monash Micromon Genomics) of the isolated plasmids. The mutant P_2_′ K Hyp1 fragment was released from pJET1.2/blunt using *XhoI* and *NcoI* and ligated into pEF‐Hyp1‐Nluc‐mDH‐FL to replace the wildtype Hyp1 fragment.

Generation of the Hyp1‐Nluc‐mDH‐FL plasmids with a truncated spacer region between the PEXEL motif and the start of the Nluc gene was carried out as follows: PCRs were performed with primer 1 paired with primer 7, 8 or 9 to produce spacers of 3aa, 13aa and 51aa, respectively (Table S5). Note that primers 7, 8 and 9 produced Hyp1 PCR products that contained one additional amino acid between the last Hyp1 residue and the start Met of Nluc. The PCR products were ligated into pJET1.2/blunt and screened as above.

To express wildtype STEVOR (PF3D7_0200400) and KARHP (PF3D7_0202000) in the Nluc‐mDH‐FL reporter plasmid, synthetic leader sequences encoding the first 99aa of STEVOR and 105aa of KAHRP containing *XhoI* and *NcoI* restriction sites were first obtained as string oligos from GeneART (ThremoFisher Scientific). P_2_′ K mutations for both STEVOR and KAHRP leaders were also synthesised as above. The synthetic DNA sequences were ligated into pJET1.2/blunt and validated by sequencing prior to transfer via *XhoI* and *NcoI* sites into the pEF‐Hyp1‐Nluc‐mDH‐FL plasmid replacing the Hyp1 sequence.

To produce pEF‐STEVOR‐Nluc‐mDH‐FL reporters with truncated spacers, PCRs were performed with primers 10 & 11 and primers 10 & 12 to produce spacer fragments of 3aa, and 13aa respectively, using pEF‐STEVOR‐Nluc‐mDH‐FL as a template (Table S5). PCR products were ligated into pJET1.2/blunt and ligated into pEF‐Nluc‐mDH‐FL via *XhoI* and *NcoI* cloning sites to produce gene fusions with only one amino acid between STEVOR/KAHRP leader sequence and Nluc. To produce the pEF‐KAHRP‐Nluc‐mDH‐FL with 3aa and 13aa spacers, PCRs were performed with primers 10 & 13 and primers 10 & 14 (Table S5). The PCR products were ligated into the Nluc‐mDH‐FL reporter as per the STEVOR reporters.

### 4.3 Indirect immunofluorescence analysis

IFA was performed essentially according to Tonkin et al.^60^ where infected RBCs were settled onto a poly‐L‐lysine (Sigma, P8920) coated coverslip and fixed with 4% paraformaldehyde/0.0075% glutaraldehyde. Following fixation, the cells were permeabilised with 0.1 M glycine/0.1% Triton X‐100 for 12 min at room temperature. Coverslips were probed overnight with primary antibodies (Table S6). The cells were washed and probed with fluorescent‐labelled secondary antibodies (Alexa Fluor 594 and 488 nm) (Table S6) for 1h at room temperature. Fixed material was mounted in VECTASHIELD with DAPI and imaged on Zeiss Cell Axio‐Observer (Carl Zeiss). Image acquisition was performed with Zen Blue imaging software.

### 4.4 Quantification of Imaging Data

All Images were visualised and analysed using ImageJ/FIJI essentially according to Gabriela et al.^42^ Briefly, a region of interest (ROI) was selected by tracing the periphery of the infected RBC on the DIC channel. The parasite area (including the PV) was selected using the outermost staining of anti‐EXP2 as a guide. Fluorescence intensity within the parasite area was then recorded (parasite fluorescence; PF). Fluorescence intensity within the ROI of the same infected cell was also recorded (total fluorescence; TF). The estimated ‘%Fluorescence in Parasite’ is the PF as a percentage of the TF after both values were corrected for the background fluorescence.

### 4.5 Chemical cross‐linking of *P. falciparum* culture and immunoprecipitation

RBCs infected with the HSP101‐HA*glmS* /PEXEL‐Nluc‐mDH‐FL parasites were enriched through magnetic purification and their proteins were solubilised in 20× pellet volume of IP lysis buffer (1% Triton X‐100, 0.1% SDS, 150 mM NaCl, 10 mM Tris–HCl pH 7.4) supplemented with cOmpleteTM Protease Inhibitor Cocktail (Roche) and subjected to 2 freeze and thaw cycles. The lysate was clarified by centrifugation and incubated overnight at 4°C with anti‐HA monoclonal agarose (Sigma‐Aldrich). Following incubation, the agarose beads were washed 5× with 1 mL IP lysis buffer and the proteins were eluted with 50 μL 2× NRSB (100 mM Tris–HCl pH 6.8, 4 mM EDTA, 4% SDS, 0.01% bromophenol blue, 20% (v/v) glycerol). For Nluc IgG immunoprecipitations, the parasite lysates were incubated overnight at 4°C with 10 μg IgG‐purified anti‐Nluc antibody. Following incubation, protein A‐Sepharose 4B (Invitrogen) was added to bind the immune complexes and samples were incubated for an additional 1 h at RT. The beads were washed, and proteins eluted as above. In all cases, both input and elution fractions were reduced in 200 mM DTT at 70°C for 5–10 min prior to SDS‐PAGE separation and western blotting.

### 4.6 Western blotting

Proteins were transferred from the gels to a nitrocellulose membrane using iBlot® Blotting System (Invitrogen). The blots were blocked in 1% casein in PBS and probed with primary antibody (Table S6) diluted in the blocking buffer overnight at 4°C. The blots were washed and probed with fluorescent‐labelled (Alexa Fluor 700 and 800 nm) or horseradish peroxidase (HRP)‐labelled secondary antibodies (Table S6) in blocking buffer for 1 h at RT followed by three washes with 1× PBS. The fluorescent secondary antibodies were visualised with a LI‐COR Odyssey FC imaging system. Densitometry analysis was performed with Image Studio v. 1.0.

### 4.7 Biochemical PEXEL cleavage assays

The cleavage assay was performed as described by Hodder et al.^41^ 2 nM of *P. vivax* PMV in buffer (25 mM Tris–HCl pH 6.4 and 25 mM MES pH 6.4) was incubated with 5 μM FRET peptide substrates representing WT and mutant KAHRP, STEVOR and Hyp1 sequences (Table S2) in a total volume of 20 μL. Samples were incubated at 20°C for 20 h and measurement was carried out using Envision plate (PerkinElmer) reader (ex. 340 nm; em. 490 nm). Biochemical *P. vivax* PMV inhibitory assays (20 μL total volume) were performed using 2 nM *P. vivax* PMV in buffer (25 mM Tris–HCl and 25 mM MES, pH 6.4) with 5 μM FRET KAHRP_WT fluorogenic peptide. Assay reactions were incubated at 37°C for 2 h in the presence of peptides (10 points dose‐response, 1 in 2 dilution series starting at 100 nM) representing STEVOR and Hyp1 sequences (Table S2). Fluorescence was measured with an Envision plate (PerkinElmer) reader (ex. 340 nm; em. 490 nm). To determine the level of PMV inhibition, ‘nonlinear regression four‐parameter to fit analysis’ using Domatics software (version 5.3.1612.8630) was performed.

### 4.8 Protein solubility analysis

Protein solubility profiling was performed according to Grüring et al.^39^ 5 μL of parasite pellet (obtained through magnetic separation) was resuspended in 100 μL hypotonic buffer (5 mM Tris–HCl pH 8.0) and subjected to 1 cycle of freezing and thawing. The soluble fraction was separated from the pellet by centrifugation at 16 000 × *g* for 5 min at 4°C. The pellet was then incubated sequentially with 100 μL 0.1 M Na_2_CO_3_ pH 11.5 and 1% Triton‐X in H_2_O for 30 min at 4°C. Soluble fractions from each incubation were transferred into a new tube. Soluble fractions were mixed with 20 μL 6x NRSB (300 mM Tris–HCl pH 6.8, 12 mM EDTA, 12% SDS, 0.03% w/v bromophenol blue, 60% v/v glycerol) and the pellet was resuspended in 120 μL 1xNRSB. All samples were kept at −20°C until used.

### 4.9 Statistical analysis

Numerical data was mainly visualised using Microsoft Excel for Mac (v.15.37) and GraphPad Prism version 9.5.0. Statistical analysis was performed using GraphPad Prism version 9.5.0 unless otherwise stated.

### 4.10 Nanoluciferase protein export assays

The nanoluciferase reporter export assays were performed as per Looker et al.^48^

## AUTHOR CONTRIBUTIONS

Mikha Gabriela, Claudia B. G. Barnes, Dickson Leong, Brad E. Sleebs, Molly Parkyn Schneider, Dene R. Littler and Paul R. Gilson performed experimental work. Mikha Gabriela, Claudia B. G. Barnes and Paul R. Gilson wrote and edited the manuscript. Brendan S. Crabb, Paul R. Gilson and Tania F. de Koning‐Ward provided funding and student supervision.

## FUNDING INFORMATION

This work was funded by NHMRC Grant and NHMRC Investigator Grant. Grant/Award numbers: 1092789 and 119780521 and Australian Research Council (ARC) grant DP160102582. This work was supported by funding from the Victorian Operational Infrastructure Support Program received by the Burnet Institute. M.G. was recipient of a Deakin University Postgraduate Research Scholarship.

## CONFLICT OF INTEREST STATEMENT

The authors declare no conflicts of interest.

### PEER REVIEW

The peer review history for this article is available at https://www.webofscience.com/api/gateway/wos/peer-review/10.1111/tra.12922.

## Supporting information


**Data S1:** Supporting Information

## Data Availability

All relevant data are within the manuscript and its Supporting Information files.
