# Supplementary material for: Sequence elements within the PEXEL motif and its downstream region modulate PTEX‐dependent protein export in Plasmodium falciparum
Source: Traffic. 2023 Nov 5;25(1):e12922. doi: 10.1111/tra.12922 (PMC10952997; doi:10.1111/tra.12922)
Supplement: Supplementary file 1 — Data S1: Supporting Information [file TRA-25-0-s001.pdf]

Supplementary Table 1. List of published P_2' mutants.

Mutated to		PEXEL Protein ID	Original PEXEL Sequence	Mutant PEXEL Sequence	PMV Cleavage	N-Acetylation	Export / Localization	Notes	Reference
Non Polar, aliphatic, Small Amino Acids	A (Alanine)	PfHRPII (PF3D7_0831800)	RLLH**E** (RLLY**E**) ; RLLYE is the Author's lab strain PfHRPII PEXEL sequence	RLLY**A**	Yes	No Information	Exported/ RBC and Parasite (see notes)	AA 1-40 of PfHRPII + GFP ; Increased intracellular protein relative to the native RLLYE ; recombinant RLLYA-GFP exhibited 7-fold decrease in interaction with PI(3)P	Bhattacharjee,*et al*.,2012 ; Bhattacharjee,*et al*.,2012
		STEVOR (No PlasmoDB ID)	RLLA**Q**	RLLA**A**	No Information	No Information	Blocked / PV	AA 1-80 of STEVOR + GFP	Przyborski,*et al.,* 2005
		STEVOR (PF3D7_0631900)	RLLA**Q**	RLLA**A**	Yes	Yes	Exported / RBC	AA 1-80 of STEVOR + GFP	Deil & Lanzer, 2014
					No Information	No Information	Exported / RBC	AA 1-63 of STEVOR + REX2 TM Domain + GFP	Gruring,*et al.* ,2012
		GBP130 (PF3D7_1016300)	RILA**E**	RILA**A**	No Information	No Information	Blocked / PV and Parasite	AA 1-99 of GBP130 + YFP	Marti,*et al* .,2004
					Yes	Yes	Blocked / PV and ER (Parasite)	AA 1-99 of GBP130 + YFP	Boddey,*et al* .,2009
					No Information	No Information	Exported / RBC	AA 1-106 of GBP130 + REX2 TM Domain + GFP	Gruring,*et al* .,2012
				**AAAAA** (Quadruple Mutation)	No	No Information	Blocked / Parasite cytoplasm or PV	AA 1-99 of GBP130 + YFP	Boddey,*et al* .,2009
		KAHRP (PF3D7_0202000)	RTLA**Q**	**ATAAA** (Triple Mutation)	No Information	No Information	Blocked /PV and Parasite	AA 1-69 of KAHRP + GFP	Marti,*et al* .,2004
					No	No Information	Blocked / PV and ER	AA 1-69 of KAHRP + GFP	Boddey,*et al* .,2009
				RTLA**A**	Yes	No Information	Blocked / ER (Early Trophs) and PV (Late Trophs)	AA 1-69 of KAHRP + GFP	Boddey,*et al* .,2009
					Yes	No Information	Exported / RBC	AA 1-69 of KAHRP + GFP	Tarr,*et al* .,2013
					Yes	Yes	Exported / RBC	AA 1-69 of KAHRP + GFP	Deil & Lanzer, 2014
		REX3 (PF3D7_0936300)	RQLS**E**	RQLS**A**	Yes	Yes	Exported / RBC	AA 1-61 of REX3 + GFP ; Capsid Protease (No PMV Version) of AA 1-61 of REX3 + GFP ; No difference in export with the Wildtype AA 1-61 REX3-GFP	Tarr,*et al* .,2013
		PfEMP3 (PF3D7_0201900)	RSLA**Q**	RSLA**A**	Yes	No Information	Exported/ RBC and Puncta in parasite	AA 1-82 of PfEMP3 + GFP (Less efficient PMV Cleavage ; High MW species present) ; Capsid Protease (No PMV Version) of AA 1-82 of PfEMP3 (No high MW species) ; Export reduced by apprx. 10% relative to wildtype*	Tarr,*et al* .,2013
		RESA (PF3D7_0102200)	RNLYG**E**	RNLYG**A**	Yes	No Information	Exported / RBC	AA 1-117 of RESA + GFP ; Export reduced by apprx. 20% relative to wildtype ; *resa* promoter	Boddey,*et al* .,2013
				**ANAYGA** (Triple Mutation)	No	No Information	Blocked / ER	AA 1-117 of RESA + GFP ; Export reduced by apprx. 95% relative to wildtype ; *resa* promoter	Boddey,*et al* .,2013
		PF3D7_0532600	RILKQ	**LNA**KA (Quadruple Mutation)	No Information	No Information	Blocked /Puncta in parasite	GFP-tagged protein	Hiller, *et al.* , 2004
		CP1 (PBANKA_1246600)	RILA**Y**	RILA**A**	No Information	No Information	Exported / RBC	Full Length PbCP1 + GFP ; EF1a Promoter	Haase,*et al* .,2013
	G (Glycine)	REX3 (PF3D7_0936300)	RQLS**E**	RQL**YG** (Double Mutation made to mimic RESA's PEXEL)	Yes (reduced efficiency)	No	Blocked / PV	AA 1-61 of REX3 + GFP ; Capsid Protease (No PMV Version) of AA 1-61 of REX3 + GFP ; Calmodulin Promoter ; Export reduced by apprx. 95-100% relative to wildtype*	Tarr,*et al* .,2013
				RQL**NG** (Double Mutation)	Yes	No Information	Blocked / PV	AA 1-61 of REX3 + GFP ; Export reduced by apprx. 95-100% relative to wildtype*	Tarr,*et al* .,2013
		PfEMP3 (PF3D7_0201900)	RSLA**Q**	RSL**YG** (Double Mutation made to mimic RESA's PEXEL)	Yes (reduced efficiency)	No Information	Blocked/ PV and Puncta in parasite	AA 1-82 of PfEMP3 + GFP (Less efficient PMV Cleavage ; High MW species present) ; Capsid Protease (No PMV Version) of AA 1-82 of PfEMP3 ; Export reduced by apprx. 75% relative to wildtype*	Tarr,*et al* .,2013

Table 1 continued.

Positively Charged,Basic Amino Acids	R (Arginine)	REX3 (PF3D7_0936300)	RQLSE	RQLSR	Yes (reduced efficiency)	No Information	Blocked / ER (Early Trophs) and PV (Late Trophs)	AA 1-61 of REX3 + GFP ; (Less efficient PMV Cleavage ; High MW species present) ; Export reduced apprx. 95-100% relative to wildtype*	Tarr,*et al*.,2013
		STEVOR (PF3D7_0631900)	RLLAQ	RLLAR	Yes	Yes	Blocked / PV	AA 1-80 of STEVOR + GFP ; Export reduced by apprx. 45% relative to wildtype	Deil & Lanzer, 2014
	K (Lysine)	STEVOR (PF3D7_0631900)	RLLAQ	RLLAK	No Information	No Information	Blocked / PV	AA 1-80 of STEVOR + GFP ; Export reduced byapprx. 95-100% relative to wildtype*	Deil & Lanzer, 2014
	H (Histidine)	STEVOR (PF3D7_0631900)	RLLAQ	RLLAH	No Information	No Information	Exported / RBC	AA 1-80 of STEVOR + GFP	Deil & Lanzer, 2014
Polar, Amide-containing Amino Acids	N (Aspargine)	STEVOR (PF3D7_0631900)	RLLAQ	RLLAN	Yes	Yes	Exported / RBC	AA 1-80 of STEVOR + GFP	Deil & Lanzer, 2014
Non Polar, Hydrophobic Amino Acids	M (Methionine)	STEVOR (PF3D7_0631900)	RLLAQ	RLLAM	No Information	No Information	Exported / RBC	AA 1-80 of STEVOR + GFP	Deil & Lanzer, 2014
Aromatic Amino Acids	Y (Tyrosine)	STEVOR (PF3D7_0631900)	RLLAQ	RLLAY	No Information	No Information	Exported / RBC	AA 1-80 of STEVOR + GFP	Deil & Lanzer, 2014
	W (Tryptophan)	STEVOR (PF3D7_0631900)	RLLAQ	RLLAW	No Information	No Information	Exported / RBC	AA 1-80 of STEVOR + GFP	Deil & Lanzer, 2014
Polar, Nucleophilic Amino Acids	S (Serine)	STEVOR (PF3D7_0631900)	RLLAQ	RLLAS	No Information	No Information	Exported / RBC	AA 1-80 of STEVOR + GFP	Deil & Lanzer, 2014
Negatively Charged, Acidic Amino Acids	D (Aspartic Acid)	STEVOR (PF3D7_0631900)	RLLAQ	RLLAD	No Information	No Information	Exported / RBC	AA 1-80 of STEVOR + GFP	Deil & Lanzer, 2014
	E (Glutamic Acid)	STEVOR (PF3D7_0631900)	RLLAQ	RLLAE	No Information	No Information	Exported / RBC	AA 1-80 of STEVOR + GFP	Deil & Lanzer, 2014

* Tarr, et al, 2013 and Deil & Lanzer, 2014 Paper expressed export level as a fraction of protein exported relative to the total signal, while Boddey et al, 2013 expressed export level as reduction relative to WT. To enable reader to directly compare the degree of export inhibition with Boddey, et al, 2013 paper, the value presented here has all been converted to % reduction relative relative to the WT .

References

1. Bhattacharjee S, Speicher KD, Stahelin RV, Speicher DW, Haldar K. PI(3)P-independent and -dependent pathways function together in a vacuolar translocation sequence to target malarial proteins to the host erythrocyte. *Molecular and Biochemical Parasitology.* 2012;185(2):106-113.
2. Bhattacharjee S, Stahelin Robert V, Speicher Kaye D, Speicher David W, Haldar K. Endoplasmic Reticulum PI(3)P Lipid Binding Targets Malaria Proteins to the Host Cell. *Cell.* 2012;148(1–2):201-212.
3. Przyborski JM, Miller SK, Pfahler JM, et al. Trafficking of STEVOR to the Maurer's clefts in *Plasmodium falciparum*-infected erythrocytes. *EMBO J.* 2005;24(13):2306-2317.
4. Deil S, Lanzer M. *New insights into PEXEL-mediated protein export in Plasmodium falciparum: the role of N-terminal acetylation*: Department für Infektiologie Heidelberg, Universität Heidelberg; 2014.
5. Grüring C, Heiber A, Kruse F, et al. Uncovering Common Principles in Protein Export of Malaria Parasites. *Cell Host & Microbe.* 2012;12(5):717-729.

6. Boddey JA, Moritz RL, Simpson RJ, Cowman AF. Role of the *Plasmodium* Export Element in Trafficking Parasite Proteins to the Infected Erythrocyte. *Traffic.* 2009;10(3):285-299.
7. Tarr SJ, Cryar A, Thalassinos K, Haldar K, Osborne AR. The C-terminal portion of the cleaved HT motif is necessary and sufficient to mediate export of proteins from the malaria parasite into its host cell. *Molecular Microbiology.* 2013;87(4):835-850.
8. Boddey JA, Carvalho TG, Hodder AN, et al. Role of plasmepsin V in export of diverse protein families from the *Plasmodium falciparum* exportome. *Traffic.* 2013;14(5):532-550.
9. Hiller NL, Bhattacharjee S, van Ooij C, et al. A host-targeting signal in virulence proteins reveals a secretome in malarial infection. *Science.* 2004;306(5703):1934-1937.
10. Haase S, Hanssen E, Matthews K, Kalanon M, de Koning-Ward TF. The exported protein PbCP1 localises to cleft-like structures in the rodent malaria parasite *Plasmodium berghei*. *PloS One.* 2013;8(4):e61482.

Supplementary Table 2. List of synthesised DNA fragments and peptides used in this study.

Sequence No.	Sequence name	Sequences (5'-3' / N-C)	Comments
**DNA fragments**			
1	STE49aa_WT	GGGCTCGAGAGATCTATGAAAATGTATTATCTTAAAATGTTATTGTTT<br>ACCTTTTTAATAAATACATTAGTAGCACGACATTATGAAAATTTTGTA<br>AATAACCATTATAATGTAAGTCTCATTCAAAACAAGACCAAAAGAGT<br>AACTATAAAATCAA**GACTTTTAGCACAA**ACCCAAATCCACAATCCG<br>CATTATCATAATGATCCAGAACTCAAAGAGATAATTGATAAAATGAA<br>CGAGGAAGCAATCAAAAAATACCAACAAACTCATGATCCATATAAA<br>CAATTGAAAGAAGTAGTAGAAAAGAATGGAGCTAGTGCATCCGCTA<br>CCATGGAAA	Used as a base to make WT, 49aa, 13aa, & 3aa STEVOR-Nluc-mDHFR-3xFLAG expression plasmid
2	STE49aa_K	GGGCTCGAGAGATCTATGAAAATGTATTATCTTAAAATGTTATTGTTT<br>ACCTTTTTAATAAATACATTAGTAGCACGACATTATGAAAATTTTGTA<br>AATAACCATTATAATGTAAGTCTCATTCAAAACAAGACCAAAAGAGT<br>AACTATAAAATCAA**GACTTTTAGCAAAA**ACCCAAATCCACAATCCG<br>CATTATCATAATGATCCAGAACTCAAAGAGATAATTGATAAAATGAA<br>CGAGGAAGCAATCAAAAAATACCAACAAACTCATGATCCATATAAA<br>CAATTGAAAGAAGTAGTAGAAAAGAATGGAGCTAGTGCATCCGCTA<br>CCATGGAAA	Used as a base to make P5 Lys STEVOR-Nluc-mDHFR-3xFLAG expression plasmid
3	KAH49aa_WT	GGGCTCGAGAGATCTATGAAAAGTTTTAAGAACAAAAATACTTTGAG<br>GAGAAAGAAGGCTTTCCCTGTTTTTACTAAAATTCTTTTAGTCTCTTTT<br>TTAGTATGGGTTTTGAAGTGCTCTAATAACTGCAATAATGGAAACGG<br>ATCCGGTGACTCCTTCGATTTCAGAAATAAG**AGAACTTTAGCACAAA**<br>AGCAACATGAACACCATCACCACCATCACCATCAACATCAACACCAA<br>CACCAAGCTCCACACCAAGCACACCACCATCATCATCATGGAGAAGT<br>AAATCACCAAGCACCACAGGTTCACCAACAAGTACATGGTCAAGAC<br>GCTAGTGCATCCGCTAACCATGGAAA	Used as a base to make WT, 49aa, 13aa, & 3aa KAHRP-Nluc-mDHFR-3xFLAG expression plasmids
4	KAH49aa_K	GGGCTCGAGAGATCTATGAAAAGTTTTAAGAACAAAAATACTTTGAG<br>GAGAAAGAAGGCTTTCCCTGTTTTTACTAAAATTCTTTTAGTCTCTTTT<br>TTAGTATGGGTTTTGAAGTGCTCTAATAACTGCAATAATGGAAACGG<br>ATCCGGTGACTCCTTCGATTTCAGAAATAAG**AGAACTTTAGCAAAAA**<br>AGCAACATGAACACCATCACCACCATCACCATCAACATCAACACCAA<br>CACCAAGCTCCACACCAAGCACACCACCATCATCATCATGGAGAAGT<br>AAATCACCAAGCACCACAGGTTCACCAACAAGTACATGGTCAAGAC<br>GCTAGTGCATCCGCTAACCATGGAAA	Used as a base to make pEF P5 Lys STEVOR-Nluc-mDHFR-3xFLAG expression plasmid
5	Hyp1 (MAE)-His-SmBiT	GAATTCGCCACCATGGCTGAATATAAAGACACCCTGCAGATCAAAGT<br>GGAACAGAAATCCCTGAGAGACTATGTGAACAACGACAGATACAAT<br>AACGTGAATACCAACGACTACACATCTTATAAGGATAAGGGCGAGCA<br>GTTCAATGATACCATTTGTGTTGTTGATCTCGAGGGAGGAGGAGGAA<br>GCGGAGGAGGAGGCAGCTCCGGTCACCATCATCACCACCATCATCAC<br>CATCATGGCGGCGGCGGCAGCGGAGGGGGAGGGAGCAGCGGGGTGA<br>CTGGCTACCGACTGTTTGAAGAAATCCTGTAATCTAGA	Inserted into the pUC57 plasmid
**Peptides**			
6	KAHRP_WT	DABCYL-RNKRTLAQKQ-E-EDANS	PEXEL cleavage assays
7	KAHRP_Double A	DABCYL-RNKATAAQKQ-E-EDANS	PEXEL cleavage assays
8	Hyp1_WT	DABCYL-G-KIRLLTEYKD-E-EDANS	PEXEL cleavage assays
9	Hyp1_K	DABCYL-G-KIRLLTKYKD-E-EDANS	PEXEL cleavage assays
10	Hyp1_A	DABCYL-G-KIRLLTAYKD-E-EDANS	PEXEL cleavage assays
11	STEVOR_WT	DABCYL-G-KSRLLAQTQI-E-EDANS	PEXEL cleavage assays
12	STEVOR_K	DABCYL-G-KSRLLAKTQI-E-EDANS	PEXEL cleavage assays
13	STEVOR_A	DABCYL-G-KSRLLAATQI-E-EDANS	PEXEL cleavage assays

Supplementary Table 3. Size estimates of WT and P$_2$' Lys Nluc-mDH-FL proteins*.

Leader Sequence	PEXEL P5 Mutation	Processing	Predicted Size (kDa)	Measured Size (Average size ± SD, kDa)
Hyp1	WT	Full Length	59.9	65.8 ± 1.4 (n=3)
		PEXEL Cleaved	52.5	51.1 ± 1.6 (n=8)
	K	SP (CIY-ES)	56.1	58.1 ± 1.2 (n=10)
		PEXEL Cleaved	52.5	49.2 ± 1.7 (n=6)
STEVOR	WT	Full Length	58.4	64.5 ± 1.9 (n=3)
		PEXEL Cleaved	52.2	49.9 ± 1.9 (n=3)
	K	Full Length	58.4	64.5 ± 1.9 (n=3)
		SP (LVA-RH)	55.9	53.8 ± 1.7 (n=3)
		PEXEL Cleaved	52.2	49.5 ± 1.7 (n=3)
KAHRP	WT	Full Length	58.8	65.1 ± 1.2 (n=3)
		PEXEL Cleaved	52.3	49.4 ± 0.8 (n=3)
	K	Full Length	58.8	64.8 ± 1.4 (n=3)
		SP (LKC-SN)	54.7	51.4 ± 1.2 (n=3)
		PEXEL Cleaved	52.3	49.5 ± 1.2 (n=3)

* The theoretical size of each protein was calculated using ProtParam (ExPASy). Signal peptidase cleavage sites were determined via SignalP5.0. Immunoblot of anti-FLAG was used to semi-quantitatively measure the protein sizes relative to the relative to the Precision Plus™ All Blue protein ladder (BioRad) migration. n, number of biological replicates. SD, standard deviation. Note that PEXEL P5 Mutation is P$_2$' mutation.

Supplementary Table 4. Size estimates of 52aa, 13aa, and 3aa Hyp1-Nluc-mDH-FL proteins.

Leader Sequence	PEXEL P5 Mutation	Processing	Predicted Size (kDa)	Measured Size (Average size ± SD, kDa)
		SP (CIY-ES)	56.1	
	WT (52aa)	PEXEL Cleaved	52.5	50.4 ± 0.9 (n=5)
Hyp1		SP (CIY-ES)	51.8	
	13aa	PEXEL Cleaved	48.2	45.8 ± 0.3 (n=5)
		SP (CIY-ES)	50.7	
	3aa	PEXEL Cleaved	47.1	44.4 ± 0.3 (n=5)

Note: The theoretical size of each protein was simulated using ProtParam (ExPASy). Signal peptidase cleavage site was determined using SignalP5.0. To obtain the empirical size of each construct, immunoblots of anti-FLAG was used to semi-quantitatively measure the protein migration relative to the Precision Plus™ All Blue protein ladder (BioRad) as a standard. n, number of biological replicates. SD, standard deviation. PEXEL P5 Mutation is P_2’ mutation.

Supplementary Table 5. List of oligonucleotide primers used in this study.

Primer No.	Primer name	Sequences (5' – 3')
1	Hyp1_1F	TGCTTATAAATAAATAAAAATTTTATAAAACTCGAGCAAAATGA
2	Hyp1_2F	TGCTAGCAGGTTATTAACAAAATATAAAGACACATTACA
3	Hyp1_3R	TGTAATGTGTCTTTATATTTTGTTAATAACCTGCTAGCA
4	Hyp1_4R	TCGAGTGTGAAGACCATGGTATCAACAACA
5	Hyp1_5F	TGCTAGCAGGTTATTAACAGCATATAAAGACACATTACA
6	Hyp1_6R	TGTAATGTGTCTTTATATGCTGTTAATAACCTGCTAGCA
7	Hyp1_3aaR	CCATGGATTCTGTTAATAACCTGCTAGCATTAAAATGTCT
8	Hyp1_13aaR	CCATGGTTTGTTCTACTTTAATTTGTAATGTGTCT
9	Hyp1_52aaR	CCATGGCATCAACAACACAAATGGTATCATTAAATTGCT
10	STEV_1F	GCTTATAAATAAATAAAAATTTTATAAAACTCGAGAGATCTATGA
11	STEV_3aaR	CCATGGATTGTGCTAAAAGTCTTGATTTTATAGTTAC
12	STEV_49aaR	CCATGGATTCTGGATCATTATGATAATGCGGATTGT
13	KAH_3aaR	CCATGGATTGCTTTTGTGCTAAAGTTCTCTTATTTCT
14	KAH_49aaR	CCATGGAGTCTTGACCATGTACTTGTTGGT

Note: Restriction sites are underlined

Supplementary Table 6. Antibodies used in this study and their sources.

Antibody	Species	Dilution		IP	Source
		IFA	WB		
Anti-HA (monoclonal HA-7)	Mouse	1/500	1/500-1/1000		Sigma-Aldrich
Anti-HA agarose (monoclonal HA-7)	Mouse			Pre-conjugated	Sigma-Aldrich
Anti-HA (mAb)	Mouse		1/500-1/1000		WEHI monoclonal antibody facility
Anti-FLAG	Mouse		10 μg/mL		WEHI monoclonal antibody facility
Anti-FLAG (M2)	Mouse	10 μg/mL			Sigma-Aldrich
Anti-FLAG	Chicken		1/2000		Abcam
Anti-nanoluciferase (IgG purified)	Rabbit	3.7-12.5 μg/mL	3.7-12.5 μg/mL	10 μg/mL	WEHI monoclonal antibody facility
Anti-EXP2 (mAb)	Mouse	5-10 μg/mL	5-10 μg/mL		WEHI monoclonal antibody facility
Anti-HSP101	Mouse		20 μg/mL		WEHI monoclonal antibody facility
Anti-PTEX150 (r741)	Rabbit		1/500		WEHI monoclonal antibody facility
Anti-PTEX150 (r740 C-term)	Rabbit		1/1000		WEHI monoclonal antibody facility
Anti-PfERC	Rabbit	1/1000			Kind gift from Matthew Dixon and Leann Tilley
Anti-PfGAPDH	Rabbit		1/2000		Kind gift from Matthew Dixon and Leann Tilley
Anti-Rabbit IgG (H+L) Alexa Fluor Plus 488	Goat	1/2000 (1 μg/mL)			Invitrogen
Anti-Rabbit IgG (H+L) Alexa Fluor 488	Goat	1/2000 (1 μg/mL)			Invitrogen
Anti-Rabbit IgG (H+L) Alexa Fluor Plus 594	Goat	1/2000 (1 μg/mL)			Invitrogen
Anti-Mouse IgG (H+L) Alexa Fluor Plus 488	Goat	1/2000 (1 μg/mL)			Invitrogen
Anti-Mouse IgG (H+L) Alexa Fluor Plus 594	Goat	1/2000 (1 μg/mL)			Invitrogen
Anti-Mouse IgG (H+L) Alexa Fluor 594	Goat	1/2000 (1 μg/mL)			Invitrogen
Anti-Rabbit IgG (H+L) Alexa Fluor Plus 700	Goat		1/10000 (0.2 μg/mL)		Invitrogen
Anti-Rabbit IgG (H+L) Alexa Fluor Plus 800	Goat		1/10000 (0.2 μg/mL)		Invitrogen
Anti-Mouse IgG (H+L) Alexa Fluor Plus 700	Goat		1/10000 (0.2 μg/mL)		Invitrogen
Anti-Mouse IgG (H+L) Alexa Fluor 680	Goat		1/10000 (0.2 μg/mL)		Invitrogen
Anti-Mouse IgG (H+L) Alexa Fluor Plus 800	Goat		1/10000 (0.2 μg/mL)		Invitrogen
Anti-Chicken IgY (HRP)	Goat		1/10000 (0.1 μg/mL)		Abcam

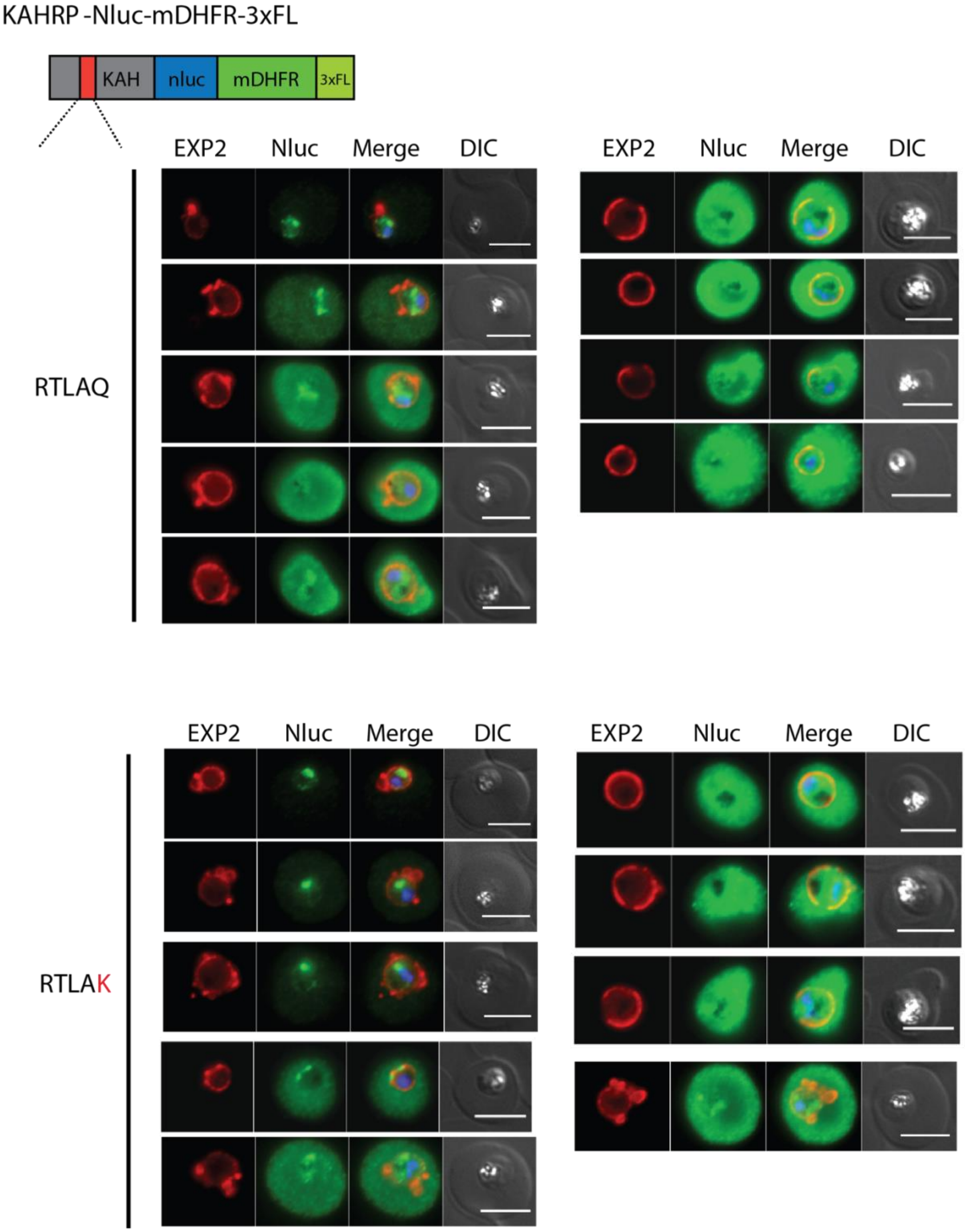

Supplementary Figure 1. Representitive immunfluorescence images of parasites expressing wildtype (RTLAQ) and P_2' K mutant (RTLAK) KAHRP-Nluc-mDH-FL reporter protein showing the full range of export phenotypes. Infected RBCs were labelled with rabbit anti-Nluc IgG and a mouse monoclonal anti-EXP2 IgG. DNA was stained with DAPI. DIC, Differential Interference Contrast. Size bar = 5 µm.

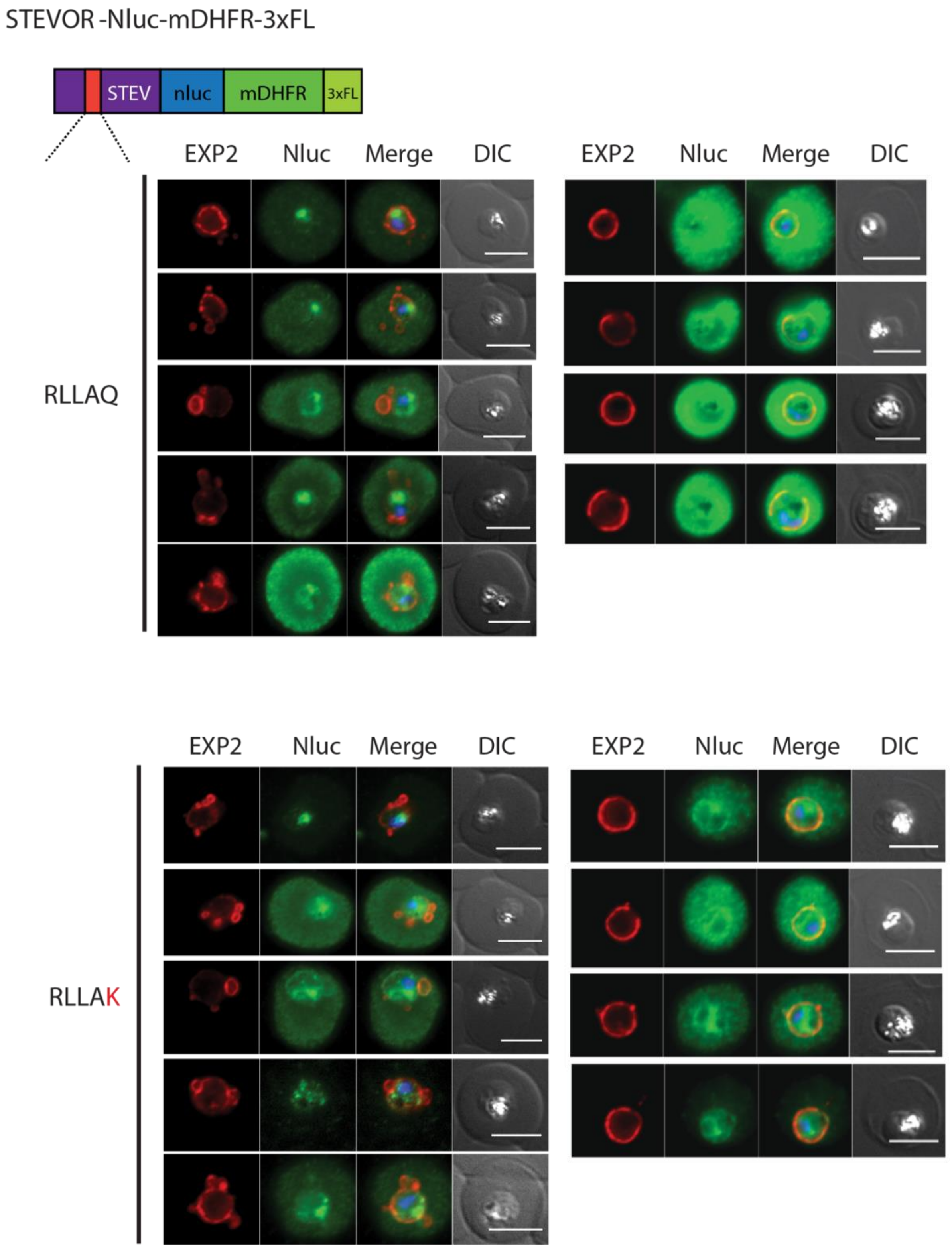

Supplementary Figure 2. Representitive immunfluorescence images of parasites expressing wildtype (RLLAQ) and P_2' K mutant (RLLAK) STEVOR-Nluc-mDH-FL reporter protein showing the full range of export phenotypes. Infected RBCs were labelled with rabbit anti-Nluc IgG and a mouse monoclonal anti-EXP2 IgG. DNA was stained with DAPI. DIC, Differential Interference Contrast. Size bar = 5 μm.

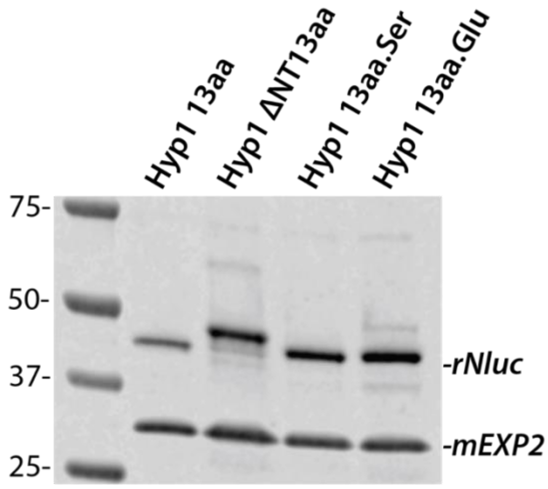

Supplementary Figure 3. Western blot analysis of Hyp1 13aa, Hyp1 ΔNT13aa, Hyp1 13aa.Ser and Hyp1 13aa.Glu parasites probed with rabbit anti-Nluc indicate the fusion proteins are of their predicted sizes and efficiently processed. Blots were also probed with an EXP2 mouse mAb as a loading control. The markers are kDa.

A. Amino acid window

WT	TEYKDTLQIKVEQ
1	TEYKDTL
2	EYKDTLQ
3	YKDTLQI
4	KDTLQIK
5	DTLQIKV
6	TLQIKVE
7	LQIKVEQ

B. Mutations

WT	TEYKDTLQIKVEQ
HP-Ala	TEAKDTAQAKAEQ
HP-Glu	TEEKDTEQEKEEQ
HP-Gly	TEGKDTGQGKGEQ
HP-Lys	TEKKDTKQKKKEQ
HP-Phe	TEFKDTFQFKFEQ
HP-Ser	TESKDTSQSKSEQ

C. Hyp1 13aa mutants

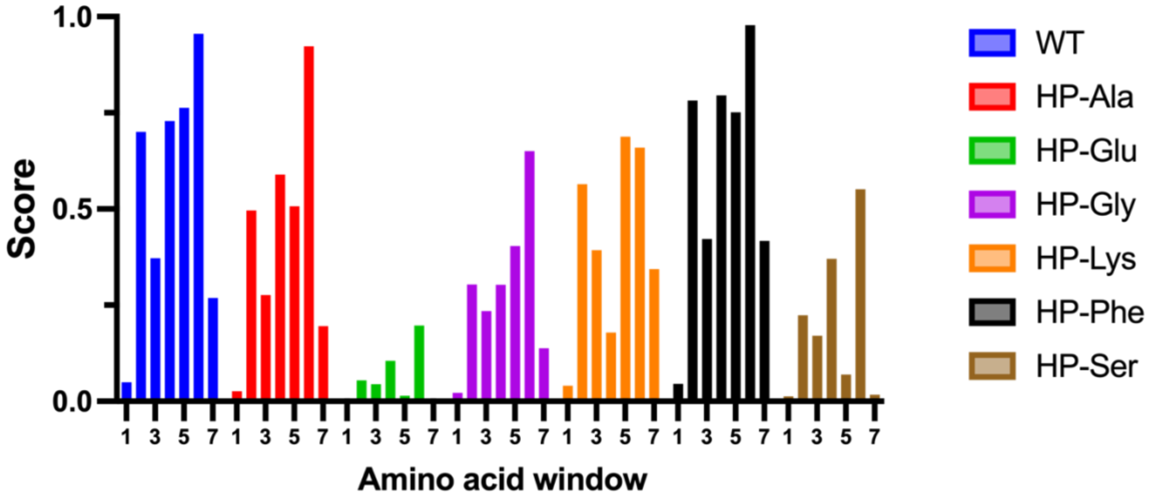

Supplementary Figure S4. Hyp1 13aa spacer mutants with hydrophobic amino acids changed to negatively charged Glu and polar Ser are predicted to most poorly bind ER HSP70 BIP. **A.** The wildtype (WT) 13 aa spacer amino acid sequence of Hyp1 downstream of the plasmespin V cleavage site and sliding window of seven amino acids scored for BIP binding (https://www.bioinformatics.wzw.tum.de/bippred/method/). **B.** Hydrophobic residues Tyr, Leu, Ile and Val were mutated to the amino acids indicated in red. **C.** Predicted BIP binding scores for each amino acid mutant displayed for the sliding window of seven amino acids as indicated in **A.**
